# Neoadjuvant concurrent chemoradiotherapy using infusional gemcitabine in locally advanced rectal cancer: A phase II trial

**DOI:** 10.1002/cam4.4590

**Published:** 2022-02-10

**Authors:** Shouki Bazarbashi, Mahmoud A. Elshenawy, Ahmed Badran, Ali Aljubran, Ahmed Alzahrani, Hadeel Almanea, Abdullah Alsuhaibani, Ahmed Alashwah, Mohamed Neimatallah, Alaa Abduljabbar, Luai Ashari, Samar Alhomoud, Hazem Ghebeh, Tusneem Elhassan, Nasser Alsanea, Mohammed Mohiuddin

**Affiliations:** ^1^ Section of Medical Oncology, Oncology Center King Faisal Specialist Hospital and Research Center Riyadh Saudi Arabia; ^2^ Clinical Oncology Department, Faculty of Medicine Menoufia University Shebin Elkom Egypt; ^3^ Clinical Oncology Department, Faculty of Medicine Ain Shams University Cairo Egypt; ^4^ Department of Pathology and Laboratory Medicine King Faisal Specialist Hospital and Research Center Riyadh Saudi Arabia; ^5^ Section of Radiation Oncology, Oncology Center King Faisal Specialist Hospital and Research Center Riyadh Saudi Arabia; ^6^ Oncology Center King Khaled University Hospital Riyadh Saudi Arabia; ^7^ Kasr El‐Aini Center for Clinical Oncology and Nuclear Medicine (NEMROCK), Faculty of Medicine Cairo University Cairo Egypt; ^8^ Department of Radiology King Faisal Specialist Hospital and Research Center Riyadh Saudi Arabia; ^9^ Department of Surgery King Faisal Specialist Hospital and Research Center Riyadh Saudi Arabia; ^10^ Research Center King Faisal Specialist Hospital and Research Center Riyadh Saudi Arabia

**Keywords:** neoadjuvant chemotherapy, neoadjuvant radiotherapy, rectal cancer, surgery

## Abstract

**Introduction:**

Gemcitabine is a well‐known radiosensitizer. Herein, we tested the efficacy and toxicity of preoperative concurrent infusional gemcitabine and radiotherapy in locally advanced rectal cancer.

**Patients and Methods:**

This was a phase II, single‐arm trial. Eligible patients had a diagnosis of rectal adenocarcinoma with clinical stage T3–T4 and/or nodal involvement, age ≥18 years, and no prior chemotherapy or radiotherapy. Patients received preoperative radiation at a dose of 50.4–54 Gy over 28 days with concurrent infusional gemcitabine administered at a dose of 100 mg/m^2^ over the course of 24 h weekly for 6 weeks. The primary endpoint was pathological complete response (pCR).

**Results:**

Forty patients were recruited. Only one patient did not complete therapy due to death. Eight patients did not undergo surgery, one died, two progressed to nonresectable disease, and five withdrew consent. Five patients progressed prior to surgery, with two having unresectable metastases and three having resectable liver metastases. One was found to have peritoneal metastasis during surgery. Out of the 32 patients who underwent surgery, seven achieved pCR at a rate of 20%. With a median follow‐up of 30 months, four additional patients had a distant relapse (one had a subsequent local relapse). The 3‐year event‐free and overall survival rates were 70% and 85%, respectively. The commonest preoperative grade 3–4 toxicity included lymphopenia (50%), neutropenia (41%), anemia (15%), diarrhea (12%), abdominal pain (12%), and proctitis (8%).

**Conclusion:**

Concurrent preoperative chemoradiotherapy using infusional gemcitabine for locally advanced rectal cancer achieved an encouraging degree of local control with manageable toxicity.

## Graphical abstract

Local recurrence and distant metastasis remain an unmet need in the management of locally advanced rectal cancer. Gemcitabine is known for its radiosensitizing properties. The study tested prospectively the efficacy and toxicity of concurrent gemcitabine and radiotherapy in the neoadjuvant setting for locally advanced rectal cancer and found a very low local recurrence rate but did not improve the rate of distant metastasis. The toxicity was manageable.

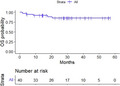

## 1 INTRODUCTION

Until recently, chemoradiotherapy using fluoropyrimidines and long‐course radiation therapy has been the most common treatment method for locally advanced rectal cancer.^1^, ^2^ This has resulted in a local recurrence rate of 4%–7%. Unfortunately, the distant metastasis rate has remained high at 19%–30%.^3^, ^4^ Several attempts have been made to improve these results. Unfortunately, the incorporation of oxaliplatin, irinotecan, biological agents (such as antivascular endothelial growth factor [bevacizumab] or antiepidermal growth factor receptors [cetuximab]), and PARP (poly[ADP]‐ribose polymerase) inhibitors in concurrent chemoradiotherapy regimens has not been found to improve the pathological complete response (pCR) or systemic recurrence rates.^4^, ^5^, ^6^, ^7^, ^8^, ^9^, ^10^, ^11^ The addition of neoadjuvant chemotherapy that is delivered prior to or after chemoradiotherapy has shown a reduction in the incidence of distant recurrences in recent studies; however, overall survival data are still pending.^12^, ^13^, ^14^, ^15^, ^16^ Several attempts have been made to reduce the recurrence rate through adjuvant chemotherapy, however, this has not been shown to improve the results primarily because of reduced compliance.^17^, ^18^, ^19^

Gemcitabine is a well‐known radiosensitizer. Several studies have shown excellent radiosensitization and acceptable toxicity for the combination of gemcitabine and radiotherapy.^20^, ^21^, ^22^, ^23^, ^24^, ^25^ Moreover, a phase I study of the combination of gemcitabine infused over 24 h and radiation in different gastrointestinal malignancies showed a maximal tolerable dose of 100 mg/m^2^ provided weekly with a clinical complete response of 50%.^26^ Similarly, a phase I/II study using biweekly gemcitabine with preoperative radiotherapy has shown manageable toxicity and an encouraging pathological response rate.^27^ Other investigators have tested the combination of 24‐h infusional gemcitabine in localized pancreatic cancer and have reported improved outcomes.^28^ The rationale for using 24‐h infusion was based on in vitro studies showing that 24‐h exposure to gemcitabine in combination with irradiation enhanced clonogenic cell killing.^29^ Based on these results, we decided to test the efficacy and toxicity of gemcitabine infused over 24 h weekly in combination with preoperative radiotherapy in patients with locally advanced rectal cancer.

## 2 PATIENTS AND METHODS

This was a single institution, open‐label, phase II trial registered at clinicaltrials.gov under the number NCT02919878. Eligible patients were required to have pathologically confirmed nonmetastatic adenocarcinoma of the rectum as well as being aged ≥18 years with a performance status of 0–2 according to the Eastern Cooperative Oncology Group (ECOG) scale. Additionally, the tumor must also have been resectable as per the surgical evaluation. Clinical stages were required to be T3 or T4 and/or lymph node‐positive disease (N1 or N2) via Magnetic Resonance Imaging (MRI) or endorectal ultrasound. In case of discrepancy in the clinical stage between MRI and endorectal ultrasound, the higher stage will be selected. Patients should have had an absolute neutrophil count (ANC) of >1,500/μl and a platelet count >100,000/μl; additionally, they should have had alanine aminotransferase (ALT), aspartate aminotransferase (AST), and alkaline phosphatase levels at <2.5 X the upper limit of normal (ULN), a bilirubin level ≤1.5 ULN, and a calculated creatinine clearance >50 ml/min via the Cockcroft–Gault formula.

Patients were excluded if they had a synchronous colonic tumor (except for a T1 lesion), had prior malignancies, had received prior chemotherapy or radiotherapy treatments, and had significant uncontrolled infections or other significant comorbidities that precluded participation in the trial.

All the patients were clinically evaluated two separate times (within 4 weeks and at 2 weeks) prior to the initiation of therapy. On both occasions, a complete blood count, a renal function chemistry profile, a hepatic function chemistry profile, and carcinoembryonic antigen (CEA) levels were requested. All the participants underwent endorectal ultrasound within 8 weeks from the start of therapy; additionally, other imaging procedures were performed, including an MRI of the pelvis via a rectal protocol, computed tomography (CT) scans of the chest, abdomen, and pelvis using oral, rectal, and intravenous contrast, and a whole‐body fluorodeoxyglucose (FDG) positron emission tomography‐CT (PET‐CT) scan.

**FIGURE 1:**
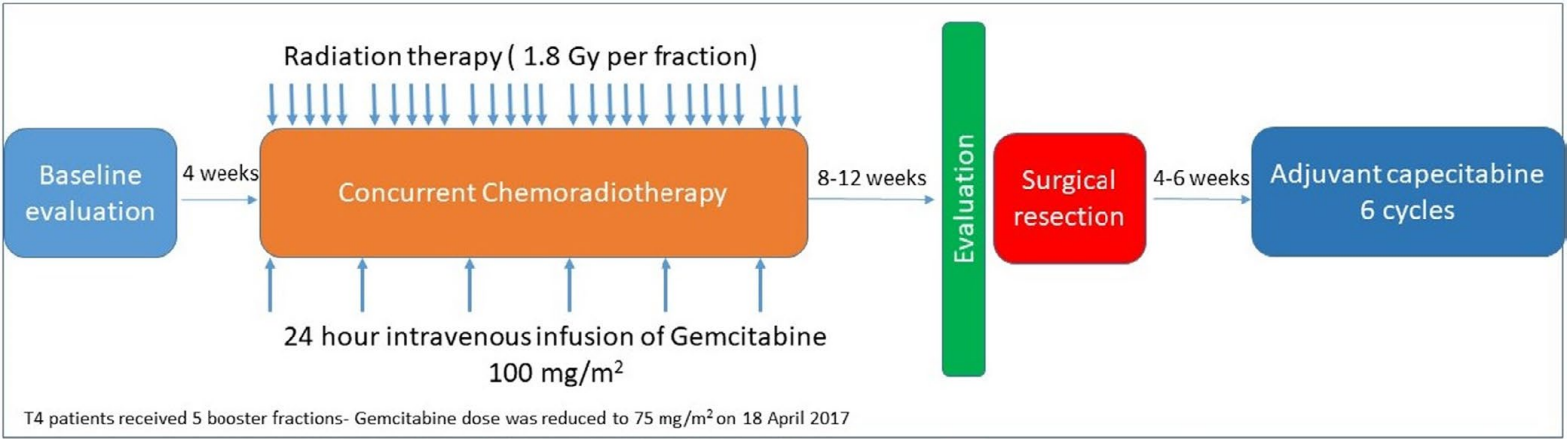
Treatment schema

Treatment consisted of neoadjuvant concurrent chemoradiotherapy, curative surgery, and adjuvant chemotherapy as illustrated in Figure 1. For neoadjuvant chemotherapy, gemcitabine was administered as a continuous intravenous infusion over 24 h through a central line at a dose of 100 mg/m^2^ weekly for 6 weeks, which was initiated on day 1 of the radiation therapy. The protocol was amended on April 18, 2017, and the dose of gemcitabine was reduced to 75 mg/m^2^, in view of the frequent grade 3 toxicities. In cases of grade 3 or 4 hematological toxicity or grade 3 nonhematological toxicity according to the Common Terminology Criteria for Adverse Events Version 4 (CTCAE V4), the gemcitabine dose was maintained until ANC was ≥1000/μl and platelets were ≥75,000/μl, or at the point when the nonhematological toxicity decreased to grade 1. Afterward, the dose was resumed at 75% of the initial dose. Gemcitabine was permanently discontinued in cases of grade 4 toxicity.

Radiation therapy was given at a dose of 1.8 Gy per fraction for 25 fractions, with a total dose of 45 Gy/25 fraction given over 5 weeks, in addition to a boost dose of 5.4 Gy for T3 and 9 Gy for T4 to a cone down volume. The total planned dose for the tumor was 50.4–54 Gy. An IMRT technique was utilized. The complete details are available in supplementary Appendix I.

All the patients were planned for surgery at 10–12 weeks following the completion of the concurrent chemoradiotherapy. Surgery findings of unresectable hepatic metastases or peritoneal seeding would preclude the radical resection of the primary tumor, unless at the discretion of the surgeon, in which cases it was indicated that it should be performed for local control and palliation. The choice of procedures involving abdominoperineal excision (APE), low anterior resection (LAR), or LAR/colo‐anal anastomosis was done at the discretion of the surgeon. The standard total mesolectal excision (TME) technique was followed for all the procedures.^30^

A full histological assessment of the resected specimen was performed in addition to the assessment of immune cell infiltration, as is detailed in supplementary Appendix I.

All the patients who underwent complete resection of the primary tumor received postoperative adjuvant chemotherapy consisting of six cycles of capecitabine at a dose of 1250 mg/m^2^ that was provided orally twice daily on days 1–14 of each cycle; the cycles were repeated every 21 days. Treatment was initiated 4–6 weeks after surgery.

Following the end of the adjuvant capecitabine treatment, patients were planned to be seen every 3 months in the first follow‐up year, after which they would be seen every 4 months for the second follow‐up year and every 6 months for years 3–5. At every visit, a complete blood count, a renal function chemistry profile, a hepatic function chemistry profile, and a CEA were requested. CT scans of the chest, abdomen, and pelvis were performed annually for 5 years and as clinically indicated. Postoperatively, patients with no proven colonic polyps were planned to undergo a full screening colonoscopy at the end of the third year and every 5 years thereafter if no polyps were identified.

The primary endpoint in this trial was to estimate the pCR rate following a neoadjuvant combined modality therapy by using weekly gemcitabine and radiation therapy in rectal cancer. Secondary endpoints were to assess the adequacy of R0 resection of the tumors following downstaging by using the previously described regimen, to estimate the incidence of hematological and nonhematological grade 3–4 toxicity with the previously described regimen, to assess the predictive value of pre‐ and post‐PET in patients treated with neoadjuvant radiotherapy and gemcitabine for locally advanced rectal cancer and to estimate the posttherapy levels of inflammatory markers (immune cells) in the tumor and compare these levels with historical controls. The last two endpoints will be presented separately.

The sample size consideration was based on the primary endpoint of pCR. Disease progression or death before surgery was considered at a lower degree than pCR (even without the surgical specimen) and was included in the denominator in cases where the pCR rate is calculated. The optimal two‐stage design to test the null hypothesis was used. After piloting the regimen on 15 patients in the first stage, the trial was terminated if two or fewer patients had pCR. If the trial progressed to the second stage, a total of 35 patients were planned. If the total number of patients responding to the treatment was ≤6, the treatment was rejected.

The analysis and early termination of the treatment (based on toxicity) were also planned. Treatment toxicity was assessed for the first 10 patients. If grade 4–5 toxicity occurred in more than 30% of cases, the protocol was terminated.

Patient characteristics were summarized using frequencies with percentages for categorical variables and medians with interquartile ranges for continuous variables. The primary endpoint (pCR) was summarized using proportion. Probabilities of OS and EFS were summarized using a Kaplan–Meier estimator with variance calculated using the Greenwood formula. OS was defined as time to death of any cause. Alive patients were censored at the last follow‐up. EFS was defined as time to disease recurrence, progression, or death. Patients who are alive and event‐free at the last follow‐up were censored. Statistical analysis was carried out using RStudio, version 1.4.1717© 2009–2021 RStudio, PBC.

Ethical consideration: Eligible patients were asked to sign informed consent prior to the performance of any trial‐related investigations. All the participating patients signed an informed consent. The trial was approved by the research ethics committee at our institution under the number RAC2141124.

## 3 RESULTS

A total of 40 patients were recruited between March 9, 2015 and October 9, 2018. The characteristics of the patients are outlined in Table 1. Of note, 10% of the patients had an ECOG performance status of two. Only 20% of the patients harbored a RAS‐wild tumor; however, in 24% of the patients, the DNA could not be amplified, and the result remained unknown. It is clear from the patient characteristics that the recruited population represented a group with poor prognostic features, due to the fact that two‐thirds of the patients (66%) had extramural vein invasion and more than half of the patients (58%) had a threatened margin.

**TABLE 1:** Characteristics of 40 patients treated with preoperative concurrent radiation and infusional gemcitabine

Characteristic	Number = 40 (%)
Age, median (range)	60.5 (38–83) years
Male sex	26 (65)
Performance status	
0	11 (27)
1	25 (63)
2	4 (10)
Histological grade	
Well differentiated	5 (13)
Moderately differentiated	30 (75)
Poorly differentiated	3 (7)
Not determined	2 (5)
RAS status	
KRAS/NRAS mutant	16 (40)
Not amplified	12 (30)
Low hemoglobin	17 (43)
Pretreatment colostomy	11 (28)
Clinical T stage	
T2	2 (5)
T3	24 (60)
T4	14 (35)
Clinical N stage	
N0	9 (23)
N1	18 (45)
N2	13 (32)
Clinical stage grouping	
IIA	3 (8)
IIIB	27 (67)
IIIC	10 (25)
EMVI	27 (67)
Threatened margin	23 (57)
Distance from anal verge	
<5 cm	16 (40)
5–10 cm	21 (53)
>10 cm	3 (7)
Tumor arising below levator Ani	11 (28)

Abbreviation: EMVI, extramural vein invasion.

Except for one patient who died before the completion of therapy, all the patients completed the radiation therapy with a median radiation dose of 5040 cGY (range: 4500–5500). The median relative dose intensity for gemcitabine was 94% (range: 46%–121%). Twenty‐three patients had their gemcitabine doses maintained for toxicity or other reasons, and 15 patients had their doses reduced.

Thirty‐seven patients underwent presurgical MRI staging. The other three patients did not undergo this procedure (one patient died and two patients withdrew consent). Presurgical MRI staging showed T1 in 13 patients, T2 in 5 patients, T3 in 11 patients, and T4 in 6 patients. In two patients, the radiologist could not determine the T and N stages, as the MRI quality was not optimal. The presurgical N stage was N0 in 17 patients, N1 in 13 patients, and N2 in 5 patients. Five patients progressed to the M1 stage at the presurgical evaluation (one with para‐aortic lymph node involvement, one with unresectable liver metastasis, and three with resectable liver metastasis). One additional patient was found to have widespread peritoneal during surgery. Radiological tumor regression grade (TRG) was grade 1 in 2 patients, grade 2 in 13 patients, grade 3 in 11 patients, grade 4 in 8 patients, and grade 5 in 1 patient. In two patients, the radiologist was unable to determine the TRG because of image quality.

**FIGURE 2:**
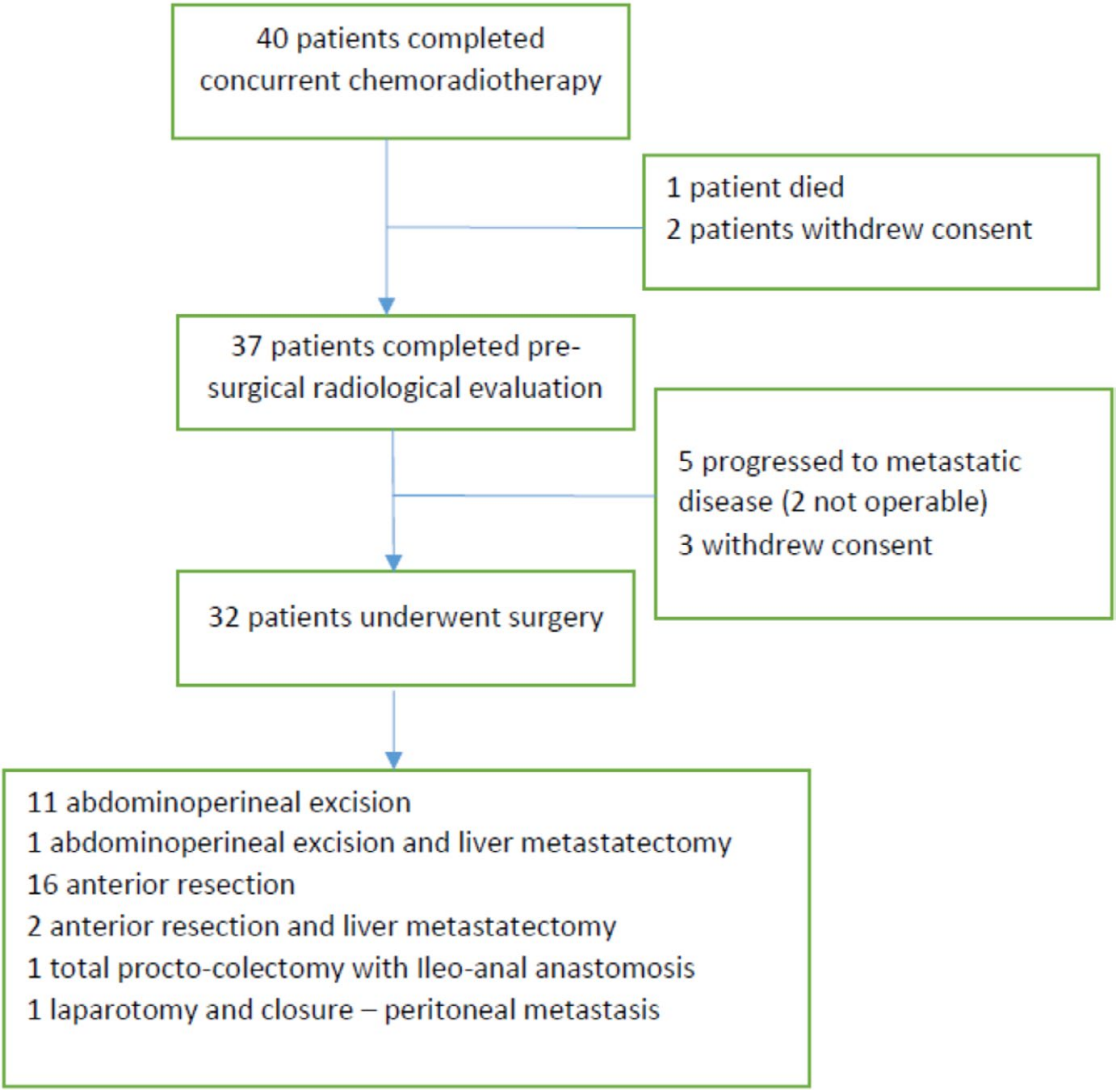
Flow diagram of 40 patients treated with preoperative concurrent radiation and infusional gemcitabine

Thirty‐two patients underwent surgery. Of the 40 enrolled patients, eight patients did not undergo surgery for various reasons; specifically, one patient died, two patients had progressions to the unresectable metastatic stage, and five patients withdrew consent. The following surgical procedures were performed: 11 abdominoperineal excisions, 16 anterior resections, 2 anterior resections with liver resection, 1 abdominoperineal excision with liver resection, 1 laparotomy, and biopsy of peritoneal metastasis without rectal excision, and 1 total proctocolectomy and ileoanal anastomosis (Figure 2).

Pathological staging showed T0 in 9 patients, T1 in 2 patients, T2 in 11 patients, T3 in 9 patients, and TX in 1 patient who was opened and then closed, as he was found to have the advanced peritoneal disease. The pathological N stage was N0 in 19 patients, N1 in 10 patients, N2 in 2 patients, and NX in 1 patient due to the previously stated reasons. The median number of lymph nodes that were removed was 14 (range: 0–32). Four patients were pathologically found to have metastatic disease (three hepatics and one peritoneal). Pathological complete responses occurred in seven patients, representing 20% (95% CI: 6.75%–33.25%) of the patients after the exclusion of patients who withdrew consent before surgery. The pathological tumor regression grade was grade 0 in 9 patients, grade 1 in 12 patients, grade 2 in 5 patients, and grade 3 in 5 patients. Details of the radiological and pathological responses are summarized in Table 2.

**TABLE 2:** Clinical and pathological downstaging following concurrent radiotherapy and infusional gemcitabine

TNM stage and TRG	Pretreatment *n* = 40 (%)	Radiological response *n* = 37 (%)	Pathological response *n* = 32 (%)
T0	0	0	9 (28)
T1	0	13 (35)	2 (6)
T2	2 (5)	5 (14)	11 (34)
T3	24 (60)	11 (30)	9 (28)
T4	14 (35)	6 (16)	0
TX	0	2 (5)	1 (3)
N0	9 (23)	17 (46)	19 (59)
N1	18 (45)	13 (35)	10 (31)
N2	13 (32)	5 (14)	2 (6)
NX	0	2 (5)	1 (3)
M0	40 (100)	35(95)	28 (88)
M1	0	2 (5)	4 (13)
TRG0	NA	0	9 (28)
TRG1	NA	2 (5)	12 (38)
TRG2	NA	13 (35)	5 (16)
TRG3	NA	11 (30)	5 (16)
TRG4	NA	8 (22)	0
TRG5	NA	1 (3)	0
TRGX	NA	2 (5)	1 (3)

Abbreviations: TNM, tumor node metastasis; TRG, tumor regression grade.

Twenty‐five patients started adjuvant chemotherapy, as per the protocol. Twenty‐four of the patients completed all six cycles of adjuvant capecitabine. One patient received only one cycle and declined to continue the treatment.

**FIGURE 3:**
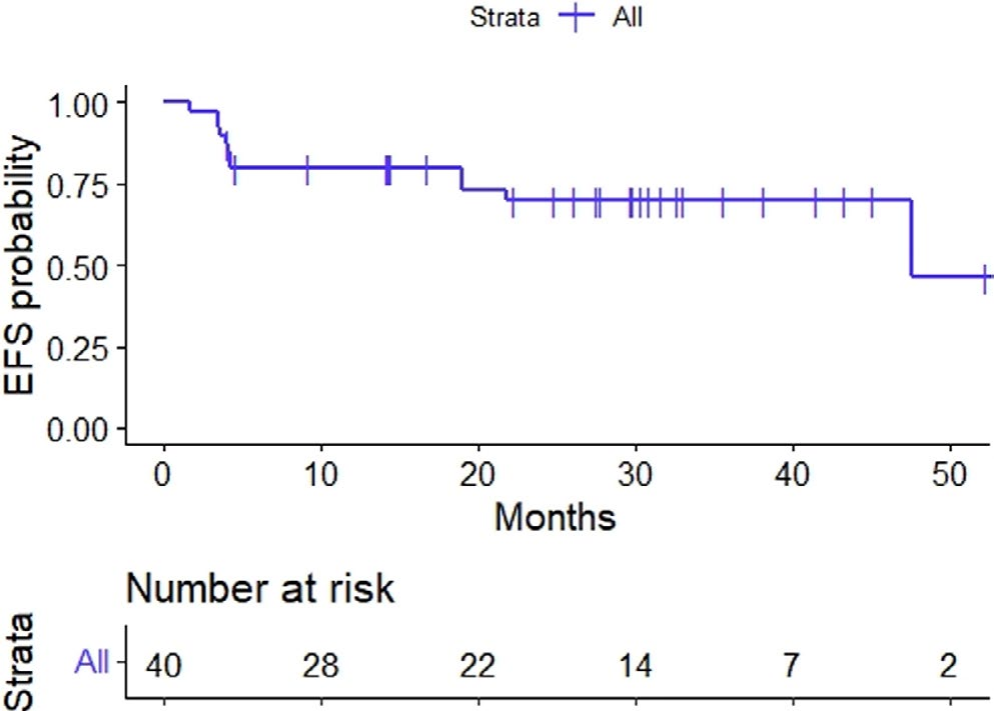
Kaplan–Meier plot for evet‐free survival in patients treated with preoperative concurrent radiation and infusional gemcitabine

**FIGURE 4:**
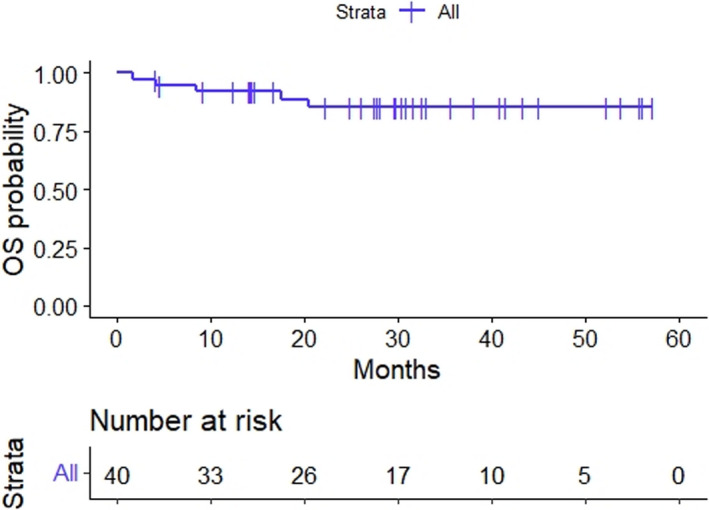
Kaplan–Meier plot for overall survival in patients treated with preoperative concurrent radiation and infusional gemcitabine

At the time of the locking of the database, the median follow‐up was 30 months (range: 4–57). A total of six patients had disease progression (both before and at the time of the surgery), and four patients had disease recurrence following surgery. Initial disease progression/recurrence was distant in all 10 patients, with 6 hepatic, 2 pulmonary, 1 peritoneal, and 1 abdominal lymph node. Only one patient had a local recurrence following distant metastasis. The 3‐year event‐free and overall survival rates were 70% (95% CI: 54.7%–85.2%) and 85% (95% CI: 73.4%–97.3%), respectively (Figures 3 and 4).

Toxicity is summarized in Table 3. Overall, 34 patients (85%) developed some form of grade 3–5 toxicity. One patient who was aged 83 years died at the end of the concurrent chemoradiotherapy at home during sleep, likely from cardiac arrest. The most common grade 3–4 hematological toxicity were leukopenia and neutropenia, which occurred at 35% and 41%, respectively. Grade 3–4 lymphopenia occurred in 50% of the patients. None of the patients developed a grade 4 nonhematological toxicity. The most common grade 3 nonhematological toxicities were diarrhea at 12%, abdominal pain at 12%, and proctitis at 8%. Eight patients (20%) developed serious adverse events that were reported within 24 h to the ethics committee.

**TABLE 3:** Acute toxicity in 40 patients who received concurrent radiation and infusional gemcitabine

Toxicity	Grade 1–2, no (%)	Grade 3, no (%)	Grade 4, no (%)	Grade 5, no (%)
Any grade 4–5 toxicity		34 (85%)		
Hematological				
Leukopenia	22 (55)	12 (30)	2 (5)	0
Neutropenia	10 (25)	13 (33)	3 (8)	0
Lymphopenia	4 (10)	13 (33)	7 (17)	0
Anemia	32 (80)	6 (15)	0	0
Thrombocytopenia	34 (85)	1 (3)	0	0
Nonhematological				
Nausea	25 (63)	0	0	0
Vomiting	9 (23)	1 (2)	0	0
Stomatitis	9 (23)	0	0	0
Diarrhea	21 (53)	5 (12)	0	0
Abdominal pain	11 (27)	5 (12)	0	0
Anorexia	8 (20)	0	0	0
Proctitis	15 (37)	3 (8)	0	0
Constipation	7 (18)	0	0	0
Fatigue	30 (75)	2 (5)	0	0
Cystitis	36 (90)	0	0	0
Skin desquamation	25 (63)	1 (2)	0	0
Hypokalemia	1 (3)	0	0	0
Hyponatremia	0	1 (3)	0	0
Hypophosphatemia	1 (3)	0	0	0
Hypomagnesemia	1 (3)	0	0	0
Sudden death	0	0	0	1 (3)

Surgical‐related complications are listed in Table 4. One patient developed peritonitis and died from septic complications. All the other patients recovered, except for one patient who developed chronic neuropathic pelvic pain requiring narcotics and gabapentin.

**TABLE 4:** Surgical‐related complications in 32 patients who underwent surgical exploration

Surgical complication	No. (%)
Wound infection	3 (8)
Pulmonary embolism	1 (3)
Septic shock/death	1 (3)
Fecal peritonitis	1 (3)
Anastomosis leak	1 (3)
Urinary obstruction	1 (3)
Postoperative temporary confusion	1 (3)
Severe pelvic pain	1 (3)
Pelvic collection	3 (8)
Pleural effusion	1 (3)
Colonic fistula	1 (3)
Postoperative 30 days mortality	1 (3)

## 4 DISCUSSION

In our patient population, rectal cancer appears to be a more aggressive disease by a stage for stage aspect than similar patients in Europe or the USA. Additionally, the response to standard preoperative chemoradiotherapy using capecitabine is less than that reported in Western studies. In our patient population, the combination of capecitabine, and radiotherapy yielded a pCR rate of 6%; additionally, with the combination of capecitabine, cetuximab and radiotherapy, the pCR rate was 12%.^30^, ^31^ This study was designed to use a novel approach to chemosensitization by using infusional gemcitabine. This study was conducted under strict clinical protocols and met its primary endpoint, with seven patients (20%) achieving pCR. In general, the combination of chemotherapy with preoperative radiotherapy aimed to elicit tumor downstaging, reduce local recurrence, and eliminate micrometastasis. The study confirmed the radiosensitizing effect of gemcitabine in rectal adenocarcinoma with significant downstaging, as was observed in the presurgical evaluation of TRG based on MRI (with 68% of patients achieving radiological TRG ≤3) and no initial local recurrence. Additionally, only one patient had local recurrence after presenting with distant failure.

Moreover, the study demonstrated the low clinical activity of gemcitabine against colorectal cancer, with all of the progressions and relapses being distant. As a single agent in colorectal cancer, the minimal clinical activity of gemcitabine has been previously reported by Chong and other researchers.^32^, ^33^ Attempts to improve this activity have been achieved through the combination of gemcitabine with 5‐fluorouracil. A review of several phase I/II trials of gemcitabine and 5‐fluorouracil in the treatment of refractory metastatic colorectal cancer was presented by Merl and colleagues.^34^ They demonstrated the encouraging efficacy and manageable toxicity of such a combination. Myelotoxicity was the most prevalent toxicity, especially when 5‐fluorouracil was given in bolus form.

Our study had similar results to the previously reported phase I/II trial of gemcitabine and hyperfractionated radiotherapy in locally advanced rectal cancer by Allal and his group.^27^ In their study, 37 patients with locally advanced rectal cancer were treated with 50 Gy of radiotherapy that was given in 2 daily fractions of 1.25 Gy per fraction over 4 weeks. Gemcitabine was given at a starting dose of 10 mg/m^2^/day in a 30‐min infusion twice per week, with a planned dose escalation of 5 mg/m^2^/day. The maximum tolerated dose was determined to be 40 mg/m^2^/day twice weekly. Forty‐seven percent of the patients had a marked pathological response, and 17% of the patients had pCR, which was similar to our study. Only one patient progressed prior to surgery, compared to five patients in our study, with one additional patient discovered to have peritoneal metastasis during surgery. In general, this was the best result (in terms of a pathological complete response) that was achieved in our patient population and was similar to other reported results of concurrent fluoropyrimidines and radiation therapy.^3^, ^4^, ^35^

The low incidence of local recurrence in the study by Allal and our study (3% local recurrence in both studies) signifies the strong radiosensitizing effect of gemcitabine and is considered to be one of the lowest local recurrence and highest local control rates in most preoperative chemoradiotherapy studies.^27^

However, the toxicity of our regimen seemed to be more than the biweekly reported regimen by Allal,^27^ with more than 30% grade 3–4 myelotoxicity and 8–12% incidence of abdominal pain, diarrhea, and proctitis. This occurred despite the amendment of the clinical trial protocol, wherein the dose of gemcitabine was reduced to 75 mg/m^2^/week (a reduction of 25%). Additionally, one of our patients died at the end of the concurrent chemoradiotherapy, although we believe that his death was unrelated because he had no major toxicity before the treatment, as well as the fact that his death occurred during sleep, which was most likely cardiac in nature.

Postoperative complications were also different in our study, with one death being secondary to septic shock, whereas 15 (47%) other patients had postoperative complications. In the trial by Allal, only eight (22%) patients (out of a total of 36 patients) had postoperative complications.^27^

In general, these complications were not different from our previously reported phase II study involving long‐course radiotherapy and capecitabine in locally advanced rectal cancer^31^ with 35% grade 3–4 diarrhea and 32% grade 3–4 proctitis. However, in that study, no perioperative death was reported, and early surgical complications included anastomotic leakage in 4 patients (13.8%), delayed wound healing in 11 patients (37.9%), and ileus in only 2 patients (6.9%).

Recently, several studies have examined the benefit of adding neoadjuvant chemotherapy in addition to chemoradiotherapy or short‐course radiotherapy. Our pathological complete response rate of 20% was intermediate between the experimental arm of the RAPIDO trial of 28% involving short‐course radiotherapy and neoadjuvant oxaliplatin‐based chemotherapy and the standard arm of 14% involving concurrent long‐course radiotherapy and capecitabine.^13^ Similar results were also observed in the UNICANCER‐PRODIGE 23 trial, wherein patients received neoadjuvant‐modified FOLFIRINOX, followed by concurrent capecitabine with long‐course radiotherapy in the investigational arm, compared with neoadjuvant concurrent capecitabine and long‐course radiotherapy in the standard arm, with pCR rates of 28% and 12%, respectively.^12^

The median follow‐up in our study was 30 months, which is considered relatively short compared with the RADIDO of 55 months and the UNICANCER‐PRODIGE 23 of 46.5 months. Our 3‐year failure‐free survival rates (compared with the investigational arms of the RAPIDO and UNICANCER‐PRODIGE 23 trials) were 75, 76.3 (reported as disease‐related treatment failure), and 76%, respectively. The 3‐year overall survival rates in our trial (compared with the other two that are listed above) were 8%5, 89.1%, and 91%, respectively.

Our study has a few limitations. The number of recruited patients is relatively small; however, this is secondary to the statistical hypothesis that was put in the trial proposal. The median follow‐up time is relatively short. We plan to do an updated follow‐up report with a longer median follow‐up duration. Five patients (representing 12.5% of the study cohort) withdrew consent; however, this is an inherent finding in many of the local studies where patients tend to travel abroad for the continuation of therapy.

## 5 CONCLUSION

The novel combination of 24‐h infusional gemcitabine and concurrent preoperative radiation in rectal cancer appears to be an effective regimen in advanced and unfavorable rectal cancer. In this study, it yielded an extremely low incidence of local recurrence and a high degree of local control. Toxicity is manageable, but distant metastasis remains a problem.

## CONFLICT OF INTEREST

The authors declare that they have no conflict of interest.

## AUTHORS' CONTRIBUTIONS

Shouki Bazarbashi: conceptualization, methodology, resources, data curation, writing—original draft preparation, reviewing, editing, and supervision; Mahmoud A. Elshenawy: data curation, writing—original draft preparation, reviewing, and editing; Ahmed Badran: data curation, writing—original draft preparation, reviewing, and editing; Ali Aljubran: methodology, resources, and data curation; Ahmed Alzahrani: methodology, resources, and data curation; Hadeel Almanea: methodology, resources, and data curation; Abdullah Alsuhaibani: methodology, resources, and data curation; Ahmed Alashwah: methodology, resources, and data curation; Mohamed Neimatallah: methodology, resources, and data curation; Alaa Abduljabbar: methodology, resources, and data curation; Luai Ashari: methodology, resources, and data curation; Samar Alhomoud: methodology, resources, and data curation; Hazem Ghebeh: methodology, resources, and data curation; Tusneem Elhassan: methodology, statistical analysis; Nasser Alsanea: writing—reviewing and editing; Mohammed Mohiuddin: conceptualization, methodology, writing—reviewing and editing.

## CLINICAL TRIAL REGISTRATION STATEMENT

This study was registered at clinicaltrial.gov under the number NCT02919878.

## ETHICAL APPROVAL STATEMENT

All study participants, or their legal guardians, provided informed written consent prior to study enrollment. The study was reviewed and approved by the hospital ethics committee under the number RAC 2141124.

## Supporting information


Appendix

## Data Availability

Data are available upon request.
