# Supplementary material for: Neoadjuvant concurrent chemoradiotherapy using infusional gemcitabine in locally advanced rectal cancer: A phase II trial
Source: Cancer Med. 2022 Feb 10;11(10):2056–66. doi: 10.1002/cam4.4590 (PMC9119355; doi:10.1002/cam4.4590)
Supplement: Supplementary file 1 — Appendix [file CAM4-11-2056-s001.doc]

Appendix

**Phase II Study of Neo-adjuvant Chemoradiotherapy using infusional Gemcitabine followed by Surgery for Locally Advanced (T3 and T4 or Node positive) Rectal Adenocarcinoma**

**RAC# 2141 124**

**NCT02919878**

Principal Investigators: Shouki Bazarbashi, MBBS (Medical Oncology)

Mohammed Mohiuddin, M.D (Radiation Oncology)

Co- Investigators: Ali AlJubran, M.D (Medical Oncology)

Ahmed Alzahrani, M.D (Medical Oncology)

Abdullah Alsuhaibani, M.D (Radiation Oncology)

Nasser al-Sanea, M.D (Colo-rectal Surgery)

Alaa Abdul Jabbar, M.D (Colo-rectal Surgery)

Samar Al Homoud, M.D (Colo-rectal Surgery)

Luai Al-Ashari, M.D (Colo-rectal Surgery)

Salahudin El Naas, M.D. (Radiology)

Hadeel Al Mana M.D. (Pathology)

Fatima Almaraiki, Pharm.D, BCOP ( pharmacy)

Edward Devol, PhD, BESC, Research Center

Ammendment 1: 3 March 2015 Approved 22 April 2015

Ammendment 2: 2 October 2015 Approved 15 November 2015

Ammendment 3: 7 February 2016 Approved 25 February 2016

Ammendment 4: 20 September 2016 Approved 18 April 2017

Ammendment 5: 31 January 2021 Approved 7 March 2021

**SchemA**

Clinical stage T3 or 4 Rectal cancer or Node + disease

**Weekly Gemcitabine* and concurrent daily XRT§**

***Gemcitabine 75 mg/m2 (24 hr infusion IV)**

**§ RT 50.4 -54 Gy (1.8 Gy/Fx)**

**Surgical resection**

**Index**

I SchemA

1. Introduction
2. Objectives
3. Patient Selection
4. Pretreatment Evaluations
5. Registration Procedures
6. Radiation Therapy
7. Drug Therapy
8. Surgery
9. Other Therapy
10. Pathology
11. Patient Assessments
12. Data Collection
13. Statistical Considerations

| References |  | |
| --- | --- | --- |
| Appendix I | | - Consent Form |
| Appendix II | | - Performance Status Scoring |
| Appendix III | | - Staging System |
| Appendix IV | | - Adverse Reporting Guideline |
| Appendix V | | - Diarrhea Diary |
| Appendix VI | | - Patient Education Sheet |
| Appendix VII | | - On study follow up table |
| Appendix VIII | | - Eligibility check list |

1. **Introduction**
   1. **Trial Synopsis**

Results of neo-adjuvant chemo-radiation for advanced rectal cancer have plateaued with pathological complete response rates of 10-20% in spite of the addition of new cytotoxic agents and/ or molecular targeted therapies. The combination of 5-FU and radiation produces effective sensitization but further improvements require assessment of other sensitizers to increase the pathological complete response rates. Gemcitabine has been used in combination with radiation for several GI cancers but few studies have examined its role in rectal cancer. This study will examine a combination of Gemcitabine and radiation as neo-adjuvant therapy in advanced rectal cancer to increase the pCR rate from our traditional 6% to > 40 %.

- 1. **Background**

Rectal cancer management remains a significant oncologic challenge worldwide. There are an estimated 36,400 new cases of cancer of the rectum in the US annually with an overall 5 year survival of 55-60 %^( 1)^. In Saudi Arabia cancers of the colon and rectum are the second most common cancer accounting for approximately 11% of all cancers and it is also the most common tumor in males. While the prognosis in patients with early tumors is excellent, patients with tumors in the distal rectum and those with increasing stage of disease (T3-T4 or N +) appear to have significantly worse outcomes. It is now well established from several studies that Neoadjuvant therapy is the preferred approach to treatment of locally advanced rectal cancers.

Adjuvant chemoradiotherapy using infusional 5-fluorouracil was established as the standard of care for stage II and III rectal cancer as it improve local recurrence and overall survival (27). The meta-analysis of the RCTs showed a significant reduction in the risk of death (17%) among patients undergoing postoperative chemotherapy as compared to those undergoing observation (HR=0.83, CI: 0.76-0.91). At a later stage, pre-operative concurrent chemoradiotherapy using 5-fluorouracil and radiation showed equal survival but better toxicity profile than post operative chemoradiotherapy in phase III German trial (28), which established the pre-operative approach as the standard of care. Recently, the NSABP R-04 trial (29) established that the oral fluoropyrimidine, capecitabine was as effective as continuous infusion 5-fuorouracil when combined with pre-operative radiotherapy without the need for central line and infusion pumps and was considered the new standard of care for the pre-operative chemotherapy in locally advanced rectal cancer.

In addition to down staging, a complete pathological response following preoperative chemo-radiation has improved as compared to radiation alone and ranges from 10-30% (2-4). Survival of patients achieving a complete response is dramatically better ranging from 85-100% even in initially advanced cancers.(5-7). In a phase II trial of neoadjuvant chemoradiotherapy using capecitabine for patients with rectal cancer at King Faisal Specialist Hospital & Research Centre, the pCR rate was only 6%. This may be related to late diagnosis, large tumor size and or poor tolerance of patients to aggressive neo-adjuvant chemo radiation (8). Recent approaches to neo-adjuvant chemo-radiation for advanced rectal cancer have included the use of additional cytotoxic drugs and /or biological agent (8-13), however, results of treatment appear to have plateaued with pathological complete response rates stagnant at 10-20% and little or no additional benefit from the added agents except an increase in toxicity.() A new strategy for combined chemo-radiation is therefore required and forms the basis for this proposal.

- 1. **Rational for combining Gemcitabine and Radiation**

Gemcitabine (2'2'Difluro-2- deoxycytadine) is a fluorine substituted analog of cytarabine ^(15)^. It has demonstrated antitumor activity in a number of murine tumor models and in human tumor xenografts^(16,17)^. It has been shown to decrease the intracellular deoxyribose nucleotide pools and increase the radiosensitivity of cells in-vitro. The initial work by Lawrence et al ^(18)^ showed that cells exposed to non-cytotoxic concentration of Gemcitabine for 24 hours and then treated with radiation, resulted in substantially increased cell kill. The dose of Gemcitabine required for radiosensitization was much lower than the therapeutic dose. In clinical practice the optimal mode for concomitant delivery of Gemcitabine and radiation still remains to be determined. A phase I study with Gemcitabine delivered as a 24 hour infusion to patients with locally advanced or metastatic non-small cell lung cancer reported a Maximum Tolerated Dose (MTD) of 180 mg/m2^(19)^

Although the mechanisms of radiosensitization are unclear, recent laboratory work from University of Michigan suggests that it lowers the threshold for radiation induced apoptosis ^(18)^. Pharmacological studies have shown that increased levels of intracellular active triphosphate metabolites can be achieved by prolonging the infusion time while holding the dose rate constant. Studies from University of Nebraska have reported the MTD of 1500mg/m2 for continuous infusion Gemcitabine given as a single agent at 10mg/m2/min infusion rate for non-hematologic malignancies ^(20)^. In a report from Netherlands ^(21)^ a two-center phase I study of Gemcitabine was undertaken in patients with advanced refractory solid tumors using a once every 2 weeks schedule. Fifty-two patients were entered into the study at 14 different dose levels starting at 40mg/m2 and escalated to 5700mg/m2. Weekly evaluation for toxicity was performed. The MTD for once 2 weekly infusion was 5700mg/m2. The dose limiting toxicity was neutropenia. It was concluded that a 2 weekly schedule was not appropriate dosing schedule for Gemcitabine. The University of Michigan trials in head and neck cancers, found that doses of Gemcitabine far below the MTD of the drug when used along with radiation therapy significantly potentiate the toxicity of treatment ^(22)^. In a study of 16 patients with advanced head and neck cancers treated with radiation and Gemcitabine given at 300mg/m2/week, 2/8 patients developed grade 4 toxicity indicating that the dose of 300mg/m2/week was significantly above the MTD of the drug. This study also demonstrated that the dose of Gemcitabine to be used for radiosensitization is significantly lower than the level used when it is administered as a cytotoxic single agent. It also demonstrated that the response rates to the combination of Gemcitabine and radiation were outstanding. Three months after completing therapy, seven of the eight patients who received 300mg/m2/week had pathological CR.

A phase I study from UNC/Wake Forest using Gemcitabine as a 30-minute infusion administered biweekly in patients with unresectable pancreatic cancer has suggested 40mg/m2 as the MTD ^(23)^. In this study the doses had been escalated from 20mg/m2 biweekly to 60mg/m2 biweekly. The dose limiting toxicities were diarrhea, neutropenia, thrombocytopenia and nausea. Some other recent studies also seem to indicate that Gemcitabine and 5FU can be given together and combined with radiation without untoward toxicity. At the Dana Farber Institute ^(24)^, a phase I study was conducted to assess the MTD of weekly Gemcitabine, given as a 24-hour continuous infusion together with infusional 5FU and Radiation , in patients with localized unresectable pancreatic adenocarcinoma. Radiation was delivered using standard 180 cGy daily fractions / 5 days per week to a total dose of 50.4 Gy. At an initial Gemcitabine dose level of 100mg/2 m /week and 5FU at 200 mg/m 2/week C.I. from days 1-7, 4/6 patients experienced dose-limiting toxicities (DLT) with grade 3 to 4 diarrhea. In addition, 3/6 patients at this dose level experienced grade 3 nausea or emesis. At the next dose level Gemcitabine at 100 mg/m2 /week and 5-FU at 200 mg/m2 /week C.I days 1-5 (the duration of 5FU was decreased), 0/3 patients had experienced a DLT. These preliminary results suggest that this strategy is feasible in patients with locally advanced pancreatic cancer. In another approach, McGinn et al ^(19)^, used radiation as a “chemosensitizer”. Full dose Gemcitabine was delivered in patients with pancreatic cancer and the radiation dose was escalated. Gemcitabine was delivered at the current recommended dose (1000 mg/m2 weekly x3 q 28 days). Radiation dose escalation was done separately in 2 groups of pts; those with the duodenum in the radiation field (unresectable patients, regardless of bypass status), and those in whom the duodenum was out of the RT field (resected pts or those with tumors in the tail of pancreas). The starting RT dose was 24 Gy in 15 fractions (1.6 Gy/fx). Dose escalation was achieved by increasing the fraction size, thus keeping the duration of RT at 3 weeks. Twenty-two patients were entered on this study at the first 2 dose levels (24 Gy and 27 Gy). Five pts experienced dose limiting toxicity (DLT); 4 with hematological DLT (grade 4 neutropenia or grade 3 thrombocytopenia; 2 occurring during Gemcitabine /RT, 2 during the second cycle of Gemcitabine) and 1 with duodenal ulceration (which was noted during the second cycle of Gemcitabine). In addition, 1 patient required transfusion for duodenal ulcer 3 months following completion of treatment. Three of 11 pts (27%) developed progressive disease in the RT field. The maximally tolerated dose (MTD) of RT when delivered concurrently with “full dose” Gemcitabine in patients with locally advanced, unresectable pancreatic cancer (+ metastatic disease), or in patients with resected disease with positive surgical margins or regional nodes was considered to be 36Gy. The response rate observed 4 weeks after completion of RT suggests that this may be a potentially valuable neoadjuvant regimen. In our study (25) to determine the maximum tolerated dose (MTD) of Gemcitabine as a 24-hour weekly infusion when administered concurrently with standard external beam irradiation, in patients with advanced gastrointestinal malignancies we found unacceptable toxicity at 150mg/m2. The dose was then de-escalated by 25mg/m2 and in the cohort of patients treated at 125 mg/m2; the toxicities were substantially less but still were at the upper limits of acceptable. We thus felt that the MTD of Gemcitabine is 100mg/m2 as a 24-hour infusion given weekly in conjunction with radiation. The once a week schedule has been convenient and patient compliance has been very good. The MTD achieved in our study is lower than in other studies as it was given as a 24-hr infusion rather than as bolus injection. The response rates, assessed radiographically (most of the patients were unable to undergo surgery as they were medically unfit), have been exceptional and are similar to the excellent response rates reported in head and neck trials. The clinical results with this 24 hour infusion of Gemcitabine have been good, and in a manner contrary to some of the laboratory evidence supporting biweekly, smaller dose, and short course Gemcitabine infusion for maximal radiosensitization. For patients with colo-rectal cancer a dose of Gemcitabine at 100mg/m2 with standard radiation was well tolerated with 75% of evaluable patients showing a good response and 50% of patients having a complete response. The major toxicity was diarrhea seen in 40% of patients, similar to that seen with 5 FU. In addition , a phase I-II trial of concurrent Gemcitabine and hyperfractionated radiotherapy performed in Switzerland performed on 37 patients with stage II-III rectal cancer showed a pathological response rate of 17% and minimal grade 3 or 4 toxicity. The above pathological CR rate was achieved despite that Gemcitabine was given in bolus format.

In our proposal: we will use gemcitabine in the same way reported by Mohiuddin et al phase I trial which is by weekly 24 hour continuous infusion, which gave a 50% clinical complete response. We believe it is worth testing this regimen in patients with locally advanced resectable rectal cancer

1. **Objectives**
   1. To estimate the pathologic complete response rate following neoadjuvant combined-modality therapy using weekly infusional Gemcitabine and external beam radiotherapy in locally advanced rectal cancer
   2. To assess the adequacy of R0 resection for tumors following down staging using the above regimen.
   3. To estimate the incidence of hematologic and non-hematologic grade 3-4 toxicity with the above regimens. The following distinct treatment periods will be considered: preoperatively, postoperatively, and overall for entire program.
   4. To assess the predictive value of pre and post PET in patient treated with neo-adjuvant radiotherapy and Gemcitabine in for locally advanced rectal cancer
   5. To estimate the inflammatory markers (immune cells) in the tumor post therapy and compare it with historical controls.
2. Patient selections
   1. Eligibility criteria
      1. Adenocarcinoma of the rectum without evidence of distant metastases
      2. Patient must be 18 years of age or greater
      3. Potentially resectable disease based upon surgeons evaluation
      4. Clinical stages T3 or T4a, and/ or positive nodes based upon endorectal ultrasound and/ or MRI.
      5. Absolute neutrophil count of > 1500 per microliter and platelet count > 100,000 per microliter; AST, AST and alkaline phosphatase < 2.5 X ULN, bilirubin < = 1.5 ULN, calculated creatinine clearance > 50 ml/min using Cockcroft-Gault formula:

Male: Creatinine Clearance = (140 – age) x weight/(72 X serum creatinine)

Female: Creatinine Clearance = (140 – age) x weight/(72 X serum creatinine x 0.85)

- - 1. ECOG performance status 0-2
    2. No history of other malignancies within 5 years, except non-melanoma skin cancer, in situ carcinoma of the cervix, or ductal carcinoma in situ of the breast. Previous invasive cancer permitted if disease free at least 5 years.
    3. Signed study-specific informed consent prior to enrolment
  1. Exclusion Criteria
     1. Any evidence of distant metastasis
     2. Synchronous primary colon carcinomas, except T1 lesions (full colonoscopy not required for enrollment)
     3. Extension of malignant disease to the anal canal
     4. Prior radiation therapy to the pelvis
     5. Prior chemotherapy for malignancies
     6. Pregnancy or lactation, (exclusion due to potential adverse effects of therapy). Women of childbearing potential with either a positive or no pregnancy test (serum or urine) at baseline. Women/men of childbearing potential not using a reliable and appropriate contraceptive method. (Postmenopausal women must have been amenorrheic for at least 12 months to be considered to be of non-childbearing potential.) Patients will agree to continue contraception for 30 days from the date of the last study drug administration.
     7. Serious, uncontrolled, concurrent infection(s).
     8. Participation in any investigational drug study within 4 weeks preceding the start of study treatment
     9. Clinically significant cardiac disease (e.g. congestive heart failure, symptomatic coronary artery disease and cardiac arrhythmias not well controlled with medication) or myocardial infarction within the last 12 months.
     10. Evidence of uncontrolled seizures, central nervous system disorders or psychiatric disability judged by the investigator to be clinically significant, precluding informed consent, or interfering with compliance of oral drug intake
     11. Other serious uncontrolled medical conditions that the investigator feels might compromise study participation.
     12. Major surgery within 4 weeks of the study treatment
     13. Lack of physical integrity of the upper gastrointestinal tract or malabsorption syndrome
     14. Known, existing uncontrolled coagulopathy
     15. No concurrent cimetidine allowed

1. Pre-Treatment evaluation
   1. Within one week of enrolment:
      1. Serum pregnancy test for females in child bearing age
   2. Within 2 weeks of enrolment:
      1. Complete physical examination
      2. Complete blood count and differential, Renal profile ( creatinine, electrolytes, serum glucose), hepatic profile ( ALT, AST, alkaline phosphatase, Albumin), Lactate dehydrogenase
   3. Within 8 weeks of enrolment:
      1. Endorectal ultrasound (TRUS) to determine the location of the tumor from anal verge and T and N stage
      2. CT scan of chest, abdomen and pelvis
      3. Magnetic resonance imaging (MRI) of pelvis
      4. Whole body PET scan
2. Registration procedure
   1. Patients can be registered only after pretreatment evaluation is completed and eligibility criteria are met. The Eligibility Checklist must be completed in its entirety prior to calling the study coordinator. The completed, signed and dated Checklist used at study entry must be retained in the patient’s study file
3. Treatment plan
   1. Pre-operatic chemotherapy: Gemcitabine will be administered as continuous infusion IV over 24 hours at a dose of 75 mg/m2 weekly for 6 weeks starting on day 1 of radiation therapy, preferably via central line/Infusor® pump
      1. Dose calculation will be according to actual body weight and not capped for obesity
      2. Institutional participation in chemotherapy studies will be in accordance with the Medical Oncology Quality Control guidelines
      3. Dose modifications for pre-operative chemotherapy ( Gemcitabine):

| Toxicity | Grade | Modification |
| --- | --- | --- |
| Hematological toxicity | Grade 1 or 2 | No change |
|  | Grade 3 or 4 | Hold chemotherapy until absolute neutrophil count ≥ 1000 and platelets ≥ 75,000, then resume at 75% of current dose. If not resolved to above parameters within 21 days delay discontinue chemotherapy. |
| Diarrhea | Grade 1 or 2 | No change |
|  | Grade 3 | Hold chemotherapy until symptoms resolve to grade ≤ 1, then resume at 75% of dose, If treatment is held for >14 days, remove patient from protocol therapy |
|  | Grade 4 | Discontinue chemotherapy- consider continue with radiation once patient recover to grade ≤ 1 |
| Lung toxicity | Grade 1 | No change |
|  | Grade 2 | Hold chemotherapy until symptoms resolve to grade ≤ 1, then resume at 75% of dose, If treatment is held for >14 days, remove patient from protocol therapy |
|  | Grade 3 or 4 | Discontinue chemotherapy- consider continue radiation once patient recover to ≤ grade 2 toxicity |

If therapy is held radiation will be made up to achieve planned total dose.

- - 1. Supportive therapy: All supportive therapy for optimal medical care will be given during the study period at the discretion of the attending physician*(s)* and documented on each case report forms as source documentation
    2. Growth Factors: Routine prophylactic use of G-CSF is not permitted; however, administration of G-CSF in patients with neutropenic complications is permitted at the discretion of the treating physician. Growth factors may not be used in lieu of dose modifications as specified in the protocol.
    3. Use of erythropoietin is not permitted.
  1. Post-operative chemotherapy: Postoperative therapy will consist of the standard 6 cycles of adjuvant Capecitabine at a dose of 1250 mg/m2 PO BID on days 1-14 each cycle, (cycles every 21 days) in patients who have a complete resection of rectal cancer and negative surgical margins. Postoperative therapy will begin 4-6 weeks after surgery. Patients with positive surgical margin will receive chemo according to investigator choice
  2. Radiation therapy:
     1. Radiation therapy should be initiated on a Sunday with 5 days of consecutive treatments.
     2. Intensity modulated radiation therapy ( IMRT ) may be utilized
     3. Fractionation
        1. Doses of 1.8 Gy per fraction five fractions per week will be delivered. The dose to the pelvis will be 45 Gy/25 fractions/five weeks with a boost dose of 5.4 Gy for T3 and 9 Gy for T4 to a cone down volume. The total dose to the tumor will be 50.4 -54 Gy. IMRT techniques may be utilized
     4. Modality: external beam photon radiation shall be used
     5. Energy: Megavoltage radiation shall be used, i.e., accelerator beams of energy no less than 6 MV.
     6. Total treatment dose
        1. Original pelvic treatment volume: The total dose to the PTV1 shall be 45 Gy in 25 (1.8 Gy/fx) fractions.
        2. Boost volume: The dose for the boost volume PTV2 is 5.4 Gy in 3 (1.8 Gy/fx) fractions for T3 and 9 Gy in 5 (1.8 Gy/fx) for T4 cancers for a cumulative dose within the tumor volume to the prescription point (or PTV) of 50.4 - 54 Gy
        3. Time dose consideration: The daily dose to the prescription point of original and boost volumes shall be 1.8 Gy.
           Fractionation: Treatment shall be given five days per week
     7. Localization, simulation and immobilization: A custom immobilization device (such as Alpha Cradle or vac-loc bag) for supine patients is suggested to minimize setup uncertainty. CT-based simulation (2 mm slice thickness) is required for this protocol and bowel exclusion techniques should be used when possible. Oral contrast may be used to allow more accurate visualization of the small bowel. Patients may be simulated supine or prone (if a belly board is utilized). Patients should be simulated with a full bladder
     8. Treatment planning and target volume: The definition of volumes will be in accordance with the 1993 ICRU Report #50: Prescribing, Recording and Reporting Photon Beam Therapy
        1. The gross tumor volume (GTV):is defined as all known gross disease as determined from a combination of physical exam, colonoscopy, ultrasound, CT (and MRI or PET-CT if performed)
        2. The clinical Target Volume (CTV): is defined as the GTV plus areas considered at significant risk of harboring microscopic disease. The CTV for a T3 tumor should include all gross disease (rectal and nodal) as well as the internal iliac lymph nodes and the meso-rectum (perirectal fat and the pre-sacral space). The CTV for a T4 tumor will include the same structures as for a T3 tumor but will include the external iliac lymph nodes as well
        3. The Planning Target Volume (PTV): will provide a margin around the CTV to compensate for the inter- and intra-fraction uncertainty consequent to daily setup uncertainty and to potential internal organ motion. By definition, the PTV will consist of a symmetrical 5 mm expansion around the CTV and 1 cm anteriorly at the level of the bladder. In the event that PTVs extend outside of the skin surface, the clinician should manually trim the PTV contours to be 3-5 mm inside the outer skin (unless there is direct skin involvement).

The following are guidelines for generating CTV and unified PTV:

Rectal GTV (+1.5 cm radially, +2.5 cm cranio-caudally) = CTV

Nodal GTV + 1.5 cm symmetrical expansion = CTV

Uninvolved iliac vessels + 1.0 cm = CTV (include external iliac if T4)

Pre-sacral lymphatic CTV is generated by contouring from mid S1-S5 and 8 mm tissue anterior to the anterior border of the sacral bone.

The meso-rectum and perirectal lymphatic CTV are generated by utilizing anatomic landmarks:

Posterior Border: anterior border of the sacrum and gluteus maximus

Lateral Border: ileum, piriformis and obturator muscles

Anterior Border: should overlap by 1 cm into the bladder, vagina or prostate

- - - 1. The PTV is generated by expanding all of the above structures by 0.5 cm symmetrically and 1 cm anteriorly at the level of the bladder and unifying them into one 3-dimensional volume for planning purposes
      2. Examples of contoured patients are available for review on the RTOG website at http://www.rtog.org/CoreLab/ContouringAtlases/Anorectal.aspx. These examples are an excellent resource for the contouring of normal structures as well as GTV, CTV and PTV design.
      3. PTV planning dose-volume constraints: (IMRT planning constraints):

≥ 98% of the PTV receives ≥ 93% of the prescribed dose

≤ 10% of the PTV receives ≥ 105% of the prescribed dose

≤ 5% of the PTV receives ≥ 110% of the prescribed dose

None of the PTV is to receive ≥ 115% of the prescribed dose

- - - 1. Dose homogeneity (3DCRT planning constraint): Dose is to be prescribed to an iso-dose surface that encompasses the PTV and that satisfies the dose uniformity guidelines below. The minimum dose to PTV1 and PTV2 shall be no less than 95% of the protocol specified dose for that volume
      2. Dose uniformity (3DCRT planning constraint): PTV1 and PTV2 shall both be encompassed within the iso-dose surface corresponding 95% of the prescription dose for that volume. The maximal dose should be no more than 110% of the prescription dose; the maximal volume to receive 110% of the prescription Dose should be kept below 10% of the PTV, as evaluated by dose volume histogram
      3. Critical structures ( IMRT planning constraints);

Small bowel:

No more than 180 cc above 35 Gy

No more than 100 cc above 40 Gy

No more than 65 cc above 45 Gy

No small bowel volume should receive 50 Gy

Femoral head:

No more than 40% volume above 40 Gy

No more than 25% volume above 45 Gy

No femoral head volume should receive 50 Gy

Bladder:

No more than 50% volume above 40 Gy

No more than 15% volume above 45 Gy

No more than 10% volume above 50 Gy

Unspecified tissue:

No specific constraints, however a DVH will be generated for “unspecified tissue” which consists of any tissue within the skin but not contoured as a part of any of the normal structures above and/or the PTV

- - 1. Treatment verification: A daily cone beam CT (KvCT or MVCT) scan of the treatment filed should be performed prior to the delivery of each fraction to assure accuracy of the treatment delivery
    2. Treatment modification: Uninterrupted treatment is planned. Treatment may be interrupted for acute toxicity. Chemotherapy will be held if RT is held. The specific reason*(s)* for treatment interruption will be recorded in the treatment summary. Treatment may be interrupted for grade ≥ 3 diarrhea or other grade ≥ 3 regional symptoms (skin desquamation, cystitis, and tenesmus). No modifications in dose will be made for interruptions in therapy. The patient will be examined at least once a week during the course of radiation with a CBC and platelets. RT interruption is to be minimized and is allowed only for regional symptoms
    3. Radiation Toxicity: All grade 4 or grade 5 toxicities that are attributable to radiation therapy will be recorded. Please refer to Section D of the Adverse Event Reporting Guidelines (*Appendix IV*) for the appropriate reporting procedures for radiation therapy related toxicity. All side effects of radiation therapy will be documented using the NCI CTCAE version 4.1. A copy of the CTCAE version 4.1 can be downloaded from the CTEP home page [(http://ctep.info.nih.gov).](http://ctep.info.nih.gov/)
  1. Surgery
     1. All patients will undergo surgery 10-12 weeks following the completion of concurrent radio-chemotherapy. The use and method of bowel preparation is at the discretion of the surgeon.
     2. The finding at surgery of unresectable hepatic metastases or peritoneal seeding will preclude radical resection of the primary unless at the discretion of the surgeon it is indicated for local control and palliation
     3. The choice of procedure abdomino-perineal resection (APR), low anterior resection (LAR), or LAR/colo-anal anastomosis) is at the discretion of the surgeon. *En bloc* hysterectomy, vaginectomy, and/or multi-visceral resection should be performed if felt to be indicated without violation of primary tumor mass. The ureters will be identified bilaterally and preserved
     4. Techniques for anastamosis are at the discretion of the surgeon, as are use, placement, and removal of pelvic drains. Total mesorectal excision (TME) is recommended for all procedures
     5. APR will involve resection of the rectum and mesorectum from the perineum to the sacral promontory. The distal left colon is divided with a linear stapler to prevent spillage of intraluminal contents at a minimum of 5 cm proximal to the tumor mass *in vivo* (not required to be documented *ex vivo* due to potential retraction). Closure of the perineum and use of pelvic drain(s) is recommended
     6. For a LAR, the entire left colon is mobilized, with ligation of the inferior mesenteric artery and vein. The distal left colon is divided with a linear stapler to prevent spillage of intraluminal contents at a minimum of 5 cm proximal to the tumor mass *in vivo* (not required to be documented *ex vivo* due to potential retraction). The rectum and meso-rectum will be removed with a distal rectal margin of at least 2 cm in vivo for sphincter preservation. Un-irradiated colon from outside the pelvis should be used for the anastomosis. If necessary takedown of the spleno-colic ligament should be performed to ensure adequate length to reach the planned anastomosis without tension.
     7. If a LAR/ Colo-anal anastomosis is performed the entire left colon is mobilized, with ligation of the inferior mesenteric artery and vein. The distal left colon is divided with a linear stapler to prevent spillage of intraluminal contents at a minimum of 5 cm proximal to the tumor mass *in vivo* (not required to be documented *ex vivo* due to potential retraction). A radical resection of the rectum and mesorectum to the levators (distal rectal margin of at least 2 cm *in vivo*) is performed from the abdominal incision. The rectosacral fascia is incised posteriorly to mobilize the entire rectum to the level of the anorectal ring. Using technique of Parks, the mucosa is stripped from the dentate line to just above the levators. At the level of the anorectal ring, the muscular rectal wall is divided by cautery and the specimen removed. The colon is brought into the anal canal and an anastomosis performed to the dentate line with interrupted full-thickness sutures. Un-irradiated colon from outside the pelvis should be used for the anastomosis. If necessary, takedown of the splenocolic ligament should be performed to avoid undue tension on the anastomosis. Use of pelvic drain(s) is recommended. Temporary diversion of the fecal stream (through the formation of either an ileostomy or transverse colostomy) should be performed. A petrolatum-impregnated gauze role or a Penrose drain is placed in the anal canal to prevent “side-to-side” healing, and removed 4-5 days later. The temporary ostomy should not be closed until at least 6-8 weeks after the completion of all cycles of post-operative chemotherapy. Following closure, patients should be kept on a regular diet with Metamucil twice daily and constipating agents as needed.
     8. Adequacy of bowel prep, estimated proximal and distal in vivo surgical margins, use of TME, anastomotic method, location of drains, need to takedown splenocolic ligament, and concomitant procedures should be clearly documented in the operative report
     9. The resected specimens are oriented for pathologic examination by placing a suture on the distal anterior rectal wall. The pathologist should ink the specimens, prior to fixation, for radial margin determination. Separate biopsies of unresected tissue at the closest tumor margins may be taken to rule out histologically residual tumor and submit in a separate bottle. Biopsy of suspicious areas on the peritoneum, liver or any other sites is recommended

1. Pathology
   1. Histopathological Assessment of the Resected Specimens
      1. The histopathological assessment after chemoradiation therapy of the cancer will be made to assess depth of invasion, grade, mucin production, and blood vessel, lymphatic or perineural invasion. The proximal colonic, distal colonic for low anterior resection or anal for abdominoperineal resection, and circumferential radial (deep) margins will be assessed in millimeters. Intra-operative frozen section will be used to assess the adequacy of the margins of resection at the discretion of the surgeon.
      2. The circumferential radial margin will be inked prior to fixation. The size of the specimen will be measured in length, width and thickness following fixation. After chemoradiation no grossly appreciable residual tumor may be present, and the tumor site may have a scar or ulcer. The size of the tumor, scar or ulcer will be measured in length, width and thickness in fixed condition. The pathology report will indicate the status of macroscopic or microscopic tumor at the proximal, distal or anal, and radial margins of resection. The central portion of the tumor, scar or ulcer will be serially sectioned to determine the maximum depth of cancer penetration.
      3. The pathology report will specify the presence or absence of mucin production. In a subset of treated cancers mucin production may be secondary to treatment. A cancer will be scored as mucin positive if 30% or more of the cells are producing mucin. The pathology will specify the presence or absence of signet ring cell morphology and a cancer will be considered a signet ring cell cancer if 90% or more of cells exhibit this morphology. The pathology report will report the presence or absence of colloid cancer morphology. The pathology report will specify depth of invasion of tumor in a fashion such that it is clear whether the tumor is:

Confined to the mucosa

Confined to submucosa

Confined to muscularis propria

Transmural into perirectal fat grossly or microscopically

- - 1. The pathology report will specify histological grade as follows:

Well differentiated, 76-100% of tumor forming glands

Moderately differentiated, 25-75% of tumor forming glands

Poorly differentiated or undifferentiated, less than 25% of tumor forming glands or tumors which contain foci which are undifferentiated or anaplastic, even if the majority of tumor forms recognizable glands.

- - 1. The pathology report will comment on the presence or absence of endothelial lined space invasion with cancer and the presence or absence of perineural and/or lymphatic invasion. An endothelial lined space containing both cancer and mature blood cells will be reported as blood vessel invasion. The number of lymph nodes involved by metastatic cancer and total number of lymph nodes will be recorded. Cytokeratin immunohistochemistry and/or step sections will be used to demonstrate viable tumor cells if lymph node(s) contain acellular mucin in the initial H&E section.
    2. Documentation of Tumor Response

Tumor response to high-dose preoperative radiation will be evaluated following surgical resection and pathological assessment of the specimen and categorized according to the following criteria:

Pathologic complete response: No evidence of tumor.

- - 1. Documentation and Diagnosis of Tumor Relapse

The following terminology will be used to document evidence of locally recurrent or metastatic disease:

- - - 1. Local Failure: recurrence or persistence of disease within radiation portals.
      2. Regional Failure: failure outside of treatment field on basis of direct and/or lymphatic spread to include aortic nodes.
      3. Distant Failure: includes both peritoneal seeding (PS) and distant metastasis (DM) on hematologic basis.
      4. Disease Relapse: will be documented by biopsy whenever possible, together with clinical or radiographic evidence.
      5. Progression: defined as one of the following:

Evidence of new areas of malignant disease (palpable or measurable).

Liver metastasis diagnosed by clinically significant hepatomegaly and/or positive liver scan.

Other evidence of progression, e.g., jaundice, ascites, pleural effusion- Class V, persistent sacral pain with or without x-ray verification of bone destruction, neurologic changes consistent with metastatic disease with positive brain or CT scan. includes both peritoneal seeding (PS) and distant metastasis (DM) on hematologic basis

7.1.8: Assessment of immune cell infiltration:

Formalin-fixed paraffin-embedded (FFPE) tissue blocks will be obtained from excised tumors after surgery. Hematoxylin and eosin (H&E) sections (4 µm) of the tissue blocks are available from each tissue block as routine hospital care for rectal cancer patients. An independent pathologist (HA), blinded to the treatment outcome, will score and interpret the histological tumor sections.

As adapted from Salgado et al 2015 and Loi et al 2013, scoring of lymphocyte infiltration will be done in a semi-quantitative estimation giving a 4-tier scale score. The score depends on the percentage of the total lymphocyte occupying the field based on morphology. Score 1 will be given for absent/rare TIL, defined as TIL occupying <10% of the scored field. Similarly, scores of 2 (mild), 3 (moderate), and 4 (severe) will be given for TIL occupying 10-30%, 30-50%, and >50% of the field, respectively.

In tumors with complete response, ulceration/necrosis areas will be assessed for TIL instead of areas rich in tumors in residual disease cases.

The patients in this trial will be compared to a control group who received capecitabine as neoadjuvant chemotherapy. The control group patients will be selected carefully to match as much as possible the response (partial and complete) response to treatment in order to segregate the histological response to chemotherapy from the effect on immune infiltration.

1. On study assessment and follow up
   1. Please refer to table appendix VII for on study assessment
   2. Complications of treatment will be recorded as to site and severity.
   3. The major complaint is most likely to be GI related, and documentation of this will be extremely important to help evaluate treatment complications. Treatment will be conservative whenever possible, with surgical intervention called upon only when conservative methods fail.
   4. Patients who have evidence of loco-regional failure either by scans or clinical examination will undergo exploratory laparotomy and radical resection if possible. The radical resection will be appropriate for the site of recurrence.
   5. Associated medical disease will be evaluated and treated as per accepted practice. Radiation can be administered as necessary for recurrent or metastatic disease.
   6. Following end of adjuvant capecitabine, patients will be seen every 3 months in 1st year, every 4 months for 2nd year and every 6 months for years 3-5. On every visit, patients will have CBCD, Renal profile, hepatic profile , and CEA.
   7. Post operatively patients will have CT scan of chest/abdomen and pelvis annually for 5 years and as clinically indicated.
   8. Post operatively patients with no proven colonic polyps will undergo full screening colonoscopy at end of third year and then every 3 years
2. Statistical considerations
   1. The primary endpoint in this trial is to estimate the pathologic complete response rate following neoadjuvant combined-modality therapy using weekly Gemcitabine and radiation therapy in rectal cancer
   2. Secondary endpoints are to estimate the incidence of hematologic and non-hematologic grade 3-4 toxicity with the above regimen. The following distinct treatment periods will be considered: preoperatively, postoperatively, and overall for entire program
   3. Sample size definitions: The following definitions apply:

N1 is the sample size in the first stage.

R1 is the treatment rejection number in the first stage.

PET is the probability of early termination of the study.

N is the combined sample size of both stages.

R is the combined treatment rejection number after both stages.

Ave N is the average sample size if this design is repeated many times.

Alpha is the probability of rejecting that P ≤ P0 when this is true.

Beta is the probability of rejecting that P ≥ P1 when this is true.

P0 is the response proportion of a poor treatment.

P1 is the response proportion of a good treatment

- 1. Sample size calculation: The sample size consideration is based on the pathological complete response (pCR). A disease progression or death before surgery will be considered as a less than pCR (even without surgical specimen), and will be included in the denominator where the path CR rate is calculated. The optimal two-stage design to test the null hypothesis that P ≤ 0.150 versus the alternative that P ≥ 0.300 has a probability of early termination of 0.717. If the drug is actually not effective, there is a 0.049 probability of concluding that it is (the target for this value was 0.050). If the drug is actually effective, there is a 0.194 probability of concluding that it is not (the target for this value was 0.200). After testing the drug on 15 patients in the first stage, the trial will be terminated if 2 or fewer respond. If the trial goes on to the second stage, a total of 35 patients will be studied. If the total number responding is less than or equal to 6, the treatment is rejected.
  2. Analysis plan and early Termination of Treatment based on toxicity:

Treatment toxicity will be assessed for the 1^st^ 10 patients. If greater than 30% experience grade 4 or 5 toxicity, the protocol will be stopped.

10.0 Ethical consideration and data confidentiality : Every effort will be made to protect the confidentiality of the patients. Patients will not be identified by their names or medical record numbers, instead, a patient identification number (PIN) will be assigned and the file assigning this numbers will be saved in locked cabinet or password protected computer excel file. Patients CRF will be locked in safe cabinet with no access except to investigators and clinical research coordinators.

Eligible patients will be asked to sign the informed consent prior to performing any trial related investigations on them. All participating patients will have to sign the informed consent and will be informed on the investigational nature of the study and will be explained the alternative options if they chose not to participate.

References

1. Jemel, A., Tiwari, R. C., Murray, T., et al: Cancer statistics; CA Cancer J Clin 54, 8-29, 2004.
2. Bosset JF, Calais G, Mineur L, et al, Does the addition of Chemotherapy (CT) to preoperative radiotherapy (preopRT) increase the pathological response in patients with resected rectal cancer: Report of the 22921 EORTC phase III trial. J Clin Oncol Vol 23 Page 247, 2004.
3. Rich TA, Skibber JM, Ajani JA, et al. Preoperative infusional chemoradiation therapy for stage T3 rectal cancer. Int J Radiat Oncol Biol Phy 32: 1025-1029, 1995.
4. Mohiuddin M. Regine WF, John WJ, et al: Preoperative chemoradiation in fixed distal rectal cancer: dose time factors for Pathological complete response. Int J. Radiat Oncol Biol Phys. March 1:46(4) :883-8, 2000.
5. Roh MS, Colangelo L, Wieand S, et al: Response to preoperative multimodality therapy predicts survival in patients with carcinoma of the rectum. J Clin Oncol 23:247, 2004.
6. Kaminsky-Forrett M-C, Conroy T, Luporsi E, et al: Prognostic implications of downstaging following preoperative radiation therapy for operable T3-T4 rectal cancer. Int J Radiat Oncol Phys, 42: 935-941; 1998.
7. Valentini V, Coco , et al. Does downstaging predict improved outcome after preoperative chemoradiation for extraperitoneal locally advanced rectal cancer? A long term analysis of 165 patients. Int J Radiat Oncol Phys, 53(3): 664-74. 2002
8. Shouki Bazarbashi, Mazen El-Bassiouni, Mahmoud Abdelsalam, Hussein Suody, Nasser Al Sanea, Alaa Abdul Jabbar, Mohamed Manji, Mosa Fagih, and Dahish Ajarim. A Modern Regimen of Pre-Operative Concurrent Chemo-Radiation Therapy in Locally Advanced Rectal Cancer. J Surg Oncol. 2008 Sep 1;98(3):167-74.
9. Freyer G, Bossard N, Romestaing P, et al. Addition of oxaliplatin to continuous fluorouracil, l-folinic acid, and concomitant radiotherapy in rectal cancer: The Lyon R 97-03 phase I trial. J Clin Oncol 19:2433-2438, 2001
10. Aschele C, Friso ML, Pucciarelli S, et al: A phase I-II study of weekly oxaliplatin, 5-fluorouracil continuous infusion and preoperative radiotherapy in locally advanced rectal cancer. Ann Oncol 16:1140-1146, 2005
11. Ryan DP, Niedzwiecki D, Hollis D, et al: Phase I/II study of preoperative oxaliplatin, fluorouracil, and external-beam radiation therapy in patients with locally advanced rectal cancer: Cancer and Leukemia Group B 89901. J Clin Oncol 24:2557-2562, 2006
12. Roh MS, Yothers GA, O'Connell MJ et al,: The impact of capecitabine and oxaliplatin in the preoperative multimodality treatment in patients with carcinoma of the rectum: NSABP R-04 J Clin Oncol 29: 2011 (suppl; abstr 3503)
13. Ge´ rard JP, Azria D, Gourgou-Bourgade S, et al: Comparison of two neoadjuvant chemoradiotherapy regimens for locally advanced rectal cancer: Results of the phase III trial ACCORD 12/0405-Prodige 2. J Clin Oncol 28:1638-1644, 2010.
14. Weiss C, Arnold D, Dellas K, et al. Preoperative radiotherapy of advanced rectal cancer with capecitabine and oxaliplatin with or without cetuximab: A pooled analysis of three prospective phase I-II trials. Int J Radiat Oncol Biol Phys 2010;78:472–478.
15. Hertel LW, Boder GB, Kroin JS, et al. Evaluation of the antitumor activity of gemcitabine (2',2'-difluoro-2'- deoxycytidine). Cancer Res 1990;50(14):4417-22.
16. Boven E, Schipper H, Erkelens CA, Hatty SA, Pinedo HM. The influence of the schedule and the dose of gemcitabine on the anti- tumour efficacy in experimental human cancer. Br J Cancer 1993;68(1):52-6
17. Shewach DS, Lawrence TS. Radiosensitization of human tumor cells by gemcitabine in vitro. Semin Oncol 1995;22(4 Suppl 11):68-71.
18. Lawrence T, Gemcitabine as a Radiosensitizer. Semin On Vol 22: 68-71, 1995. McGinn CJ, Shureiqi I, Robertson JM, Eckhauser FE, Smith DC, Decker DA, Neuman HK, Brown D, Strawderman M, Lawrence TS. Encouraging survival data from a phase I trial of radiation (RT) dose escalation with full dose gemcitabine (GEM) in patients with unresectable pancreatic cancer. Proc. ASCO abstract #1051 p 274a, 1999.
19. Brand R, Capadam M, Tempero M. A phase I trial of weekly gemcitabine administered as a prolonged infusion in patients with pancreatic cancer and other solid tumors. Invest new drugs 15: 4, 331-341, 1997.
20. Vermobeen JB, Guastalla JP, Hatty SR et al. Phase I study of gemcitabine using once every 2 weeks schedule. Br J Cancer 76 (11) 1489-93, 1997.
21. Eisbrach A, Sheneach DS, Usbar S et al. A phase I study of gemcitabine concurrent with radiation for advanced Head and Neck tumors: high rate of mucosal and pharynpul toxicity - Proc Am Soc Clin Oncol 16: 3689, 1997.
22. Blackstock WA, Bernard SA, Richards F, et al. Phase I Trial of Twice Weekly Gemcitabine and concurrent radiation in patients with advanced pancreatic cancer. JCO 17: 2208-2212, 1999.
23. Fuchs CS, Clark JW,Berg DT, Warshaw AL, Mamon HJ, Grossbard ML, Kulke MH, Morgan JA, Ryan DP, Zinner MJ, Osteen RT, Mayer RJ, Willett CG. Phase I trial of gemcitabine (GEM), infusional 5-Fluorouracil (FU) and radiation Therapy (RT) in patients with localized, Unresectable Pancreatic Adenocarcinoma (PAC). Proceedings of Am Soc Clin Onc (18) 284, 1999
24. Mohiuddin M, Kudrimoti M, Regine W, et al. Concurrent infusional gemcitabine and radiation in the treatment of advanced unresectable GI malignancy: a phase I/II study. J Clin Oncol 2000.
25. Jones CL, Holmgren E: An adaptive Simon Two-Stage Design for Phase 2 studies of targeted therapies Contemporary Clinical Trials 28(2007) 654-661
26. Allal A, Bieri S, et al. Pre-operative concurrent hyperfractionated radiotherapy and Gemcitabine for locally advanced rectal cancers: A phase I-II trial. The cancer Journal. 11(2):133-139; 2005
27. Sune Høirup Petersen, Henrik Harling, et al. Postoperative adjuvant chemotherapy in rectal cancer operated for cure. (Review). *The Cochrane Library*, 2012, Issue 3
28. Rolf Sauer, Heinz Becker, et al. Preoperative versus Postoperative Chemoradiotherapy for Rectal Cancer. N Engl J Med 351;17
29. Carmen Joseph Allegra, Greg Yothers. Neoadjuvant therapy for rectal cancer: Mature results from NSABP protocol R-04. J Clin Oncol 32, 2014 (suppl 3; abstr 390)

Appendix I: **CONSENT FOR RESEARCH STUDY**

**Phase II trial of neo-adjuvant combined modality therapy using Gemcitabine and surgery for locally advanced rectal cancer**

This is a clinical trial (a type of research study). Clinical trials include only patients who choose to take part. Please take your time to make your decision. Discuss it with your friends and family.

You are being asked to take part in this study because you have cancer of the rectum.

**Why is this study being done?**

The purpose of this study is to use chemotherapy medication combinations that will be given to patients with and without radiation to obtain a better result than can be achieved in the treatment of this disease. We want to find out what effects (good and bad) the treatments have on rectal cancer. This research is being done because although surgery is the standard treatment for advanced rectal cancer, there is a high rate of the cancer coming back. We want to find out if using newer types of chemotherapy ( Gemcitabine) combined with radiation and chemotherapy given before surgery and chemotherapy after surgery will help control this disease. Having chemotherapy and radiation therapy before surgery may also reduce the tumor size so that less surgery may be necessary

**How many people will take part in the study?**

About 25 people will take part in this study.

**What does the study involve?**

If you agree to participate, you will have a physical exam and your medical history taken. You will also receive radiation therapy once a day, five days per week (Saturday through Wednesday) for six weeks, and chemotherapy will be given by intravenous infusion over 24 hours every Saturday during the radiation. This will be followed by surgery which constitute removal of tumor six-eight weeks after chemotherapy treatment ends. One month following the surgery you will receive chemotherapy as pills for a period of 4 months which part of the standard therapy for rectal cancer

If you take part in this study, you will have the following tests and procedures that are part of regular cancer care and may be done even if you do not join the study. Those include physical exam, blood counts and chemistries, chest x-ray, MRI of pelvis, PET scans, examination of the bowel with a fiberoptic flexible tube, examination of the bowel with a small flexible tube with an ultrasound device and CT of abdomen and pelvis.

**How long will I be in the study?**

Treatment will last eight to nine months. You will receive radiation therapy and chemotherapy for five to six weeks, and then, you will have surgery six to eight weeks later. However, if you decide to stop participating in the study, we encourage you to talk to your regular doctor first. If you do not complete the prescribed treatment, you will still have regular checkups with your doctor for the following 5 years. You will have these checkups once every three months for two years, then once every six months for the remaining three years.

If your disease gets worse in spite of the treatment, your doctor may decide to take you off this study treatment regimen. If the side effects of the treatment are too dangerous for you, or new information about the treatment becomes available and this information suggests the treatment will be ineffective or unsafe for you, your doctor may decide to take you off the study treatment regimen. Even if you are not getting the study treatment regimen, you will still be in the follow-up part of the study.

**What are the risks of the study?**

While on the study, you are at risk for these side effects. You should discuss these with your doctor. There also may be other side effects that we cannot predict. Other drugs will be given to make side effects less serious and uncomfortable. Many side effects go away shortly after the radiation therapy and chemotherapy are stopped, but in some cases, side effects can be serious or long lasting or permanent.

Risks and side effects related to radiation therapy we are studying include:

*Very likely:*

Skin irritation, Diarrhea, Tiredness, Nausea, Temporary loss of pubic hair. For women: Sterility in pre-menopausal women. Hormones may be given orally to replace hormones normally produced by the ovaries.

*Less Likely, but Serious*:

Intestinal blockage and/or intestinal bleeding which may require surgery. For men: Permanent sterility.

*Risks Associated with Chemotherapy*:

*Very Likely*:

Lower blood counts, which can lead to risk of infection and bleeding. Loss of appetite. Nausea and/or vomiting. Weakness/fatigue. Skin rash. Loss of hair

*Less Likely*:

Headaches. Darkening of skin, nails or veins. Changes in vision (Blurring) Rash or allergic reaction Flu-like symptoms such as fever, chills and muscle aches.

*Less Likely, but Serious*:

Confusion or memory loss. Infection at the catheter entry site. Lung damage resulting in shortness of breath (which may be permanent). Blood clots. Blood pressure changes and hemorrhage may also occur.

Although rare, it is possible that treatment-related side-effects could result in death.

*Reproductive Risks*

This study may be harmful to a nursing infant or an unborn child. Sufficient medical information is not available to determine whether the study treatment administered to a pregnant woman causes significant risks to the fetus. If you are a woman able to have children and have not been surgically sterilized (tubal ligation or hysterectomy), you should have a pregnancy test before enrolling in this study. If you are unwilling to use adequate birth control measures to prevent pregnancy, you should not participate in this study. If you should become pregnant while on study, you must tell your doctor immediately.

If you are a man able to father children, the treatment you receive may risk harm to an unborn child unless you use a form of birth control approved by your doctor. If you are unwilling to use adequate birth control measures to prevent pregnancy, you should not participate in this study. If you suspect you have caused anyone to become pregnant, you must tell your doctor immediately.

**Are there benefits to taking part in the study:**

If you agree to take part in this study, there may be direct medical benefit to you. This might include improvement in the result of your therapy which may transplate to more chances of cure from your disease. We hope the information learned from this study will also benefit other patients with rectal cancer in the future.

**What other options are there?**

You may choose not to participate in this study. Other treatments that could be considered for your condition may include the following: (1) radiation therapy; (2) chemotherapy; (3) surgery; or (4) no treatment except medications to make you feel better. With the latter choice, your tumor would continue to grow and your disease would spread. These treatments could be given either alone or in combination with each other.

Your doctor can tell you more about your condition and the possible benefits of the different available treatments. Please talk to your regular doctor about these and other options.

**What about confidentiality?**

Efforts will be made to keep your personal information confidential. Records of your progress while on the study will be kept in a confidential form at this institution. You will receive no payment for taking part in this study.

Appendix II: **Performance status scoring**

**KARNOFSKY PERFORMANCE SCALE**

100 Normal; no complaints; no evidence of disease

90 Able to carry on normal activity; minor signs or symptoms of disease

80 Normal activity with effort; some sign or symptoms of disease

70 Cares for self; unable to carry on normal activity or do active work

60 Requires occasional assistance, but is able to care for most personal needs

50 Requires considerable assistance and frequent medical care

40 Disabled; requires special care and assistance

30 Severely disabled; hospitalization is indicated, although death not imminent

20 Very sick; hospitalization necessary; active support treatment is necessary

10 Moribund; fatal processes progressing rapidly

0 Dead

**ECOG PERFORMANCE SCALE**

0 : Fully active, able to carry on all predisease activities without restriction (Karnofsky 90-100).

1 : Restricted in physically strenuous activity but ambulatory and able to carry out work of a light or sedentary nature. For example, light housework, office work (Karnofsky 70-80).

2 : Ambulatory and capable of all self-care but unable to carry out any work activities. Up and about more than 50% of waking hours (Karnofsky 50-60).

3 : Capable of only limited self-care, confined to bed or chair 50% or more of waking hours (Karnofsky 30-40).

4 : Completely disabled. Cannot carry on any self-care. Totally confined to bed or chair (Karnofsky 10-20).

**Appendix III:** AJCC staging system

| **Primary tumor (T)** | | | | | |
| --- | --- | --- | --- | --- | --- |
| TX | Primary tumor cannot be assessed | | | | |
| T0 | No evidence of primary tumor | | | | |
| Tis | Carcinoma in situ: intraepithelial or invasion of lamina propria* | | | | |
| T1 | Tumor invades submucosa | | | | |
| T2 | Tumor invades muscularis propria | | | | |
| T3 | Tumor invades through the muscularis propria into pericolorectal tissues | | | | |
| T4a | Tumor penetrates to the surface of the visceral peritoneum• | | | | |
| T4b | Tumor directly invades or is adherent to other organs or structures•Δ | | | | |
| **Regional lymph node (N)** | | | | | |
| NX | Regional lymph nodes cannot be assessed | | | | |
| N0 | No regional lymph node metastasis | | | | |
| N1 | Metastasis in 1-3 regional lymph nodes | | | | |
| N1a | Metastasis in one regional lymph node | | | | |
| N1b | Metastasis in 2-3 regional lymph nodes | | | | |
| N1c | Tumor deposit(s) in the subserosa, mesentery, or nonperitonealized pericolic or perirectal tissues without regional nodal metastasis | | | | |
| N2 | Metastasis in four or more regional lymph nodes | | | | |
| N2a | Metastasis in 4-6 regional lymph nodes | | | | |
| N2b | Metastasis in seven or more regional lymph nodes | | | | |
| **Distant metastasis (M)** | | | | | |
| M0 | No distant metastasis | | | | |
| M1 | Distant metastasis | | | | |
| M1a | Metastasis confined to one organ or site (eg, liver, lung, ovary, nonregional node) | | | | |
| M1b | Metastases in more than one organ/site or the peritoneum | | | | |
| **Anatomic stage/prognostic groups** | | | | | |
| **Stage** | **T** | **N** | **M** | **Dukes** | **MAC¥** |
| 0 | Tis | N0 | M0 | - | - |
| I | T1 | N0 | M0 | A | A |
|  | T2 | N0 | M0 | A | B1 |
| IIA | T3 | N0 | M0 | B | B2 |
| IIB | T4a | N0 | M0 | B | B2 |
| IIC | T4b | N0 | M0 | B | B3 |
| IIIA | T1-2 | N1/N1c | M0 | C | C1 |
|  | T1 | N2a | M0 | C | C1 |
| IIIB | T3-T4a | N1/N1c | M0 | C | C2 |
|  | T2-T3 | N2a | M0 | C | C1/C2 |
|  | T1-T2 | N2b | M0 | C | C1 |
| IIIC | T4a | N2a | M0 | C | C2 |
|  | T3-T4a | N2b | M0 | C | C2 |
|  | T4b | N1-N2 | M0 | C | C3 |
| IVA | Any T | Any N | M1a | - | - |
| IVB | Any T | Any N | M1b | - | - |

Appendix IV: **adverse reporting guidelines**

Local regulations require that investigators report adverse events and reactions in a timely manner. This reporting improves patient care and scientific communication by providing information to the office of research affairs whereby new findings can be more widely disseminated to investigators and scientists.

A. *Definitions and Terminology*: An **adverse event** is defined as an undesirable, unfavorable or unintended sign (including an abnormal laboratory finding), symptom or disease associated with the use of a medical treatment or procedure regardless of whether it is considered related to the medical treatment or procedure. This may be a new event that was not pre-existing at initiation of treatment, a pre-existing event that recurs with increased intensity or frequency subsequent to commencement of treatment or an event, though present at the commencement of treatment, becomes more severe following initiation of treatment. These undesirable effects may be classified as “known or expected” or “unknown or unexpected”.

A **serious adverse event** is any adverse event that, at any dose, fulfills at least one of the following criteria:

- is **fatal** (results in death) (note: death is an outcome, not an event)
- is **life-threatening** (note: the term “life-threatening” refers to an event in which the patient was at risk of death at the time of the event; it does not refer to an event which could hypothetically have caused death had it been more severe).
- required patient hospitalization or prolongation of existing hospitalization (note: “inpatient hospitalization” refers to an unplanned, overnight hospitalization).
- results in persistent or significant disability/incapacity.
- is a congenital anomaly/birth defect.
- is medically significant or requires intervention to prevent one or other of the outcomes listed above.

**Known/expected events** are those that have been previously identified as having resulted from administration of the agent or treatment. They may be identified in the literature, the protocol, the consent form, or noted in the drug insert.

**Unknown/unexpected events** are those thought to have resulted from the agent, e.g. temporal relationship but not previously identified as a known effect.

*Assessment of Attribution*

In evaluating whether an adverse event is related to a procedure or treatment, the following attribution categories are utilized:

**Definite:** The adverse event is clearly related to the treatment/procedure.

**Probable:** The adverse event is likely related to the treatment/procedure.

**Possible**: The adverse event may be related to the treatment/procedure.

**Unlikely**: The adverse event is doubtfully related to the treatment/procedure.

**Unrelated**: The adverse event is clearly NOT related to the treatment/procedure.

B. *Grading of Adverse Events*: Unless specified otherwise, the NCI Common Terminology Criteria for Adverse Events (CTCAE) version 4.1 is used to grade severity of adverse events

C*. General Guidelines*: In order to assure prompt and complete reporting of adverse events and toxicity, the following general guidelines must be observed:

1. The Investigator will report to the to the Study Chair within 24 hours of discovery, the details of all unexpected severe, life-threatening (grade 4) and fatal (grade 5) adverse events if there is reasonable suspicion that the event was definitely, probably, or possibly related to protocol treatment.
2. All deaths during protocol treatment or within 30 days of completion or termination of protocol treatment regardless of attribution require telephone notification within 24 hours of discovery.
3. A written report, including all relevant clinical information and all study forms due up to and including the date of the event, will be sent to the PI.
   1. The Study Chair will take appropriate and prompt action to inform Office of research affairs (ORA)/ IRB of the SAE andof any protocol modifications and/or precautionary measures, if this is warranted.

D. *Adverse Event Reporting Related to Radiation Therapy*

1. All fatal events resulting from protocol radiation therapy must be reported by telephone to the PI within 24 hours of discovery.

2. All grade 4, (CTCAE v 4.1 ) and life-threatening events (an event, which in view of the investigator, places the patient at immediate risk of death from the reaction) that is related, possibly related or probably related to protocol treatment using non-standard fractionated radiation therapy, brachytherapy, radiopharmaceuticals, high LET radiation, and radiosurgery must be reported by telephone to PI within 24 hours of discovery. Expected grade 4 adverse events may be excluded from telephone reporting if specifically stated in the protocol.

3. All applicable data forms and if requested, a written report, must be submitted to the PI within 10 working days of the telephone call.

E. *Adverse Event Reporting Related to Systemic Anticancer Agents*

Adverse drug reactions (ADRs) are adverse events that are related to an anticancer agent and meet certain criteria: are unexpected effects of the drug or agent, or are severe (grade 3), life-threatening (grade 4), or fatal (grade 5), even if the type of event has been previously noted to have occurred with the agent.

Appendix V: **Diarrhea Diary**

**PATIENT**: Enter # bowel movements/day in excess of pretreatment # of daily bowel movement(s) Call the office for any diarrhea

| **Day of Week** | **# Bowel Movements / # Imodium pills** | **Day of Week** | **# Bowel Movements / Treatment for Diarrhea** |
| --- | --- | --- | --- |
| Sun |  | Sun |  |
| Mon |  | Mon |  |
| Tues |  | Tues |  |
| Wed |  | Wed |  |
| Thurs |  | Thurs |  |
| Fri |  | Fri |  |
| Sat |  | Sat |  |
| Sun |  | Sun |  |
| Mon |  | Mon |  |
| Tues |  | Tues |  |
| Wed |  | Wed |  |
| Thurs |  | Thurs |  |
| Fri |  | Fri |  |
| Sat |  | Sat |  |

COMMENTS______________________________________________________

NURSE/RA SIGNATURE

PATIENT’SIGNATURE

(INITIALS)__________________

Appendix IV **: Patient education sheet**

**MANAGEMENT OF DIARRHEA DURING CHEMOTHERAPY**

Patient Initials______ Patient #________

The patient was counseled regarding the potential side effects that may occur during treatment. This specifically addresses DIARRHEA MANAGEMENT.

DIARRHEA

You must call your doctor/nurse if you experience any diarrhea and follow the instructions below:

♦ Have a supply of loperamide (e.g., Imodium) at home and begin loperamide treatment for an increase in your usual number of daily bowel movements by more than 2 per day. If you experience ANY diarrhea you must call your doctor/nurse at that time so that you can be given additional instructions. You should also follow the instructions below.

Loperamide (Imodium) 2 mg tablets/ capsules should be taken as follows:

♦ 2 tablets (4mg) at the first onset of diarrhea

♦1 tablet (2mg) every 2 hours during the day until 12 hours after last loose stool ♦2 tablets (4mg) every 4 hours during the night until 12 hours after the last loose stool

♦ If diarrhea does not stop after 24 hours of loperamide call your treating physician/ nurse for further instructions

♦ Drink at least eight –8oz glasses of fluid every day.

Appendix VII: on study follow up table

| **Assessment** | **Study Entry** | **Day1 of every week of XRT** | **Day 1 of week4,6** | **Pre-Surgeryc** | **Post Op Prior to each adjuvant chemo and FU visit** |
| --- | --- | --- | --- | --- | --- |
| **Physical Exam** | **X** |  | **X** | **X** | **X** |
| **CBC diff, Platelets** | **X** | **X** |  | **X** | **X** |
| **CEA** | **X** |  |  | **X** |  |
| **Alk Phos, AST,ALT total bilirubin, creatinine, LDH** | **X** |  | **X** | **X** | **X** |
| **Serum or urine pregnancy test*** | **X** |  |  |  |  |
| **CT Scan (abd/pelvis) to include liver** | **X** |  |  | **X** | **Annually and**  **as indicated** |
| **MRI pelvis** | **X** |  |  | x | As indicated |
| **Transrectal ultrasound (Optional for T4)** | **X** |  |  |  |  |
| **Chest x-ray** | **X** |  |  |  |  |
| **Lower endoscopy** | **X** |  |  |  |  |
| **ECOG performance** | **X** |  | **X** | x |  |
| **Toxicity Assessments** | **X** |  | **X** | **X** | **X** |
| **PET Scan** | **x** |  |  | x |  |
| **Gemcitabine 100 mg/m2 IV infusion over 24 hours** |  | **X** |  |  |  |

Appendix VIII: **Eligibility check list**

|  |  | | |  |  |
| --- | --- | --- | --- | --- | --- |
| **Case #** | ___________ | | |  |  |
|  |  | | |  |  |
| _______(Y) | | 1. | Does patient have rectal adenocarcinoma? | | |
| _______(N) | | 2. | Is there tumor extension to the anal canal? | | |
| _______(Y/N) | | 3. | Is tumor fixed? (T4) | | |
| _______(Y) | |  | If no, is tumor T3 and mobile by endorectal ultrasound? | | |
| _______(Y/N/A) | | 4. | If female, is patient non-pregnant and non-lactating? | | |
| _______(>1,500) | | 5. | What is the ANC? | | |
| _______(>100K) | | 6. | What is platelet count? | | |
| _______(Y) | | 7. | Is bilirubin < 1.5 x upper limit normal limit? | | |
| _______(Y) | | 8. | Are liver and kidney functions acceptable *(*AST, AST and alkaline phosphatase < 2.5 X ULN, bilirubin < = 1.5 ULN, calculated creatinine clearance > 50 ml/min using Cockcroft-Gault formula*)?* | | |
| _______(N) | | 9. | Does patient have other serious illnesses? | | |
| _______(N) | | 10. | Did patient receive any prior chemotherapy or XRT to plevis? | | |
| _______(Y/N) | | 11. | Any prior malignancy? | | |
|  | |  | _______(Y) If yes, disease free 5 years? | | |
| _______(N) | | 12. | Any concurrent malignancy other than non-melanoma skin cancer or *in situ* cancer of cervix? | | |
| _______(N) | | 13. | Any unresected synchronous Tis or T1 colonic cancer? | | |
| _______(N) | | 14. | Is there any evidence of distant metastases? | | |
| _______(0-2) | | 15. | What is ECOG status? | | |
| _______(Y) | | 16. | Have pretreatment evaluations been completed as specified in Section x | | |
|  | |  |  | | |

**The following questions will be asked at Study Registration:**

_____________ 1. Name of institutional person registering this case?

____________(Y)2. Has the Eligibility Checklist *(above)* been completed?

____________(Y)3. Is the patient eligible for this study?

_____________ 4. Date the study-specific Consent Form was signed? *(must be prior to study entry)*

_____________ 5. Patient’s Name

_____________ 6. Verifying Physician

_____________ 7. Patient’s hospital ID Number

_____________ 8. Date of Birth

_____________ 11. Gender

_____________ 16. Treatment Start Date

_____________ 17. Medical Oncologist

_____________ 18. Clinical Stage *(T3 vs. T4)*?

| Completed by | ____________________________ | Date | _________________ |
| --- | --- | --- | --- |
